# Cuneiform Nucleus Stimulation Can Assist Gait Training to Promote Locomotor Recovery in Individuals With Incomplete Tetraplegia

**DOI:** 10.1002/ana.78026

**Published:** 2025-09-10

**Authors:** Anna‐Sophie Hofer, Lennart H. Stieglitz, Marc Bolliger, Linard Filli, Adrian Cathomen, Romina Willi, Irina Lerch, Iris Krüsi, Melina Giagiozis, Christian Meyer, Martin Schubert, Michèle Hubli, Thomas M. Kessler, László Demkó, Christian R. Baumann, Lukas Imbach, Markus F. Oertel, Andrea Prusse, Alina Kiseleva, Luca Regli, Martin E. Schwab, Armin Curt

**Affiliations:** ^1^ Department of Neurosurgery, Clinical Neuroscience Center University Hospital Zurich Zurich Switzerland; ^2^ Institute for Regenerative Medicine University of Zurich Schlieren Switzerland; ^3^ Spinal Cord Injury Center Balgrist University Hospital, University of Zurich Zurich Switzerland; ^4^ Swiss Center for Movement Analysis (SCMA), Balgrist Campus AG Zurich Switzerland; ^5^ Department of Neuro‐Urology Balgrist University Hospital, University of Zurich Zurich Switzerland; ^6^ Department of Neurology, Clinical Neuroscience Center University Hospital Zurich Zurich Switzerland; ^7^ Swiss Epilepsy Center Klinik Lengg Zurich Switzerland

## Abstract

**Objective:**

Impaired ability to induce stepping after incomplete spinal cord injury (SCI) can limit the efficacy of locomotor training, often leaving patients wheelchair‐bound. The cuneiform nucleus (CNF), a key mesencephalic locomotor control center, modulates the activity of spinal locomotor centers via the reticulospinal tract. Even with severe corticospinal damage, the widely distributed reticulospinal fibers frequently cross the lesion, and lumbosacral spinal locomotor centers remain responsive. Unilateral deep brain stimulation (DBS) of the CNF (CNF‐DBS) can increase modulatory input to sublesional locomotor centers and was shown to induce stepping and promote locomotor recovery in rodent models of severe incomplete SCI. Given the evolutionarily conserved CNF‐reticulospinal system, we hypothesize that CNF‐DBS can augment training and improve gait in humans with incomplete SCI above the lumbosacral levels.

**Methods:**

Aiming at bench‐to‐bedside translation, we investigate CNF‐DBS in non‐ambulatory patients (clinicaltrials.gov, NCT03053791). Here, we present the first 2 individuals with chronic tetraplegia who underwent 6 months of locomotor training supported by unilateral CNF‐DBS, with regular follow‐up assessments of adverse and therapeutic effects performed without and with stimulation.

**Results:**

The walking distance covered during the 6‐Minute Walking Test (6MWT) after 6 months compared to baseline served as the primary study end point, which was reached by patient 1 in the off‐condition and by patient 2 in the off‐ and the on‐condition. No serious adverse events occurred.

**Interpretation:**

We show that the CNF‐DBS was well tolerated and had therapeutic potential in the first 2 patients, and discuss the lessons learnt with resulting implementations for the next patients. ANN NEUROL 2026;99:161–177

Spinal cord injury (SCI) is a devastating event with often life‐long consequences on mobility. Therapeutic options to promote motor recovery are limited, with neurorehabilitative training playing a central role.^1^, ^2^ Brain‐controlled circuits in the lumbosacral spinal cord, the central pattern generators,^3^, ^4^ are active during locomotion and receive input from brainstem motor centers like the mesencephalic locomotor region (MLR),^5^ which is comprised of the cuneiform nucleus (CNF) and the pedunculopontine (PPN) nucleus.^6^ After incomplete SCI, spinal locomotor centers lose supraspinal input due to partially disrupted signal transmission via spared fiber tracts, disabling stepping and hindering gait training.^7^ Even with intensive rehabilitation, recovery often remains limited,^8^ leaving many patients dependent on mobility aids. Neuromodulatory strategies have been developed to facilitate supraspinal motor drive to sub‐lesional circuits, divided into spinal cord and brain stimulation.^1^ Evidence supports spinal cord stimulation as promising option for selected patients.^9^, ^10^, ^11^ At the supraspinal level, the MLR, which indirectly acts via the reticulospinal tract, has emerged as an appealing target.^12^ Particularly the CNF plays a key role in initiating and controlling locomotion,^6^ making it a candidate for deep brain stimulation (DBS).^13^, ^14^ Preclinical studies showed that CNF stimulation steers locomotion in an intensity dependent manner in intact animals,^13^, ^15^ preserving context‐specific locomotor control.^13^ In subchronic and chronic SCI rodent models, it acutely improved hindlimb movements,^13^, ^14^, ^15^ and CNF‐DBS during training significantly enhanced locomotor recovery.^13^, ^16^ Given the dispersed localization of reticulospinal fibers in the human spinal cord white matter^17^ and the predominance of anatomically incomplete SCIs,^18^, ^19^, ^20^, ^21^ some functional reticulospinal fibers are likely preserved after injury.^22^ We hypothesize that CNF stimulation can enhance motor drive via spared reticulospinal fibers in patients with incomplete SCI above the lumbosacral levels, aiding in stepping and improving activity‐based rehabilitation. To investigate feasibility, side effects, and the therapeutic potential of CNF‐DBS‐assisted training, we launched a clinical trial^23^ (https://clinicaltrials.gov, NCT03053791). Here, we report the first 2 patients with traumatic chronic SCI who underwent 6 months of CNF‐DBS‐supported rehabilitative training. The primary end point was walking distance in the 6‐Minute Walking Test (6MWT) after 6 months compared to baseline. Secondary motor and non‐motor outcomes were also assessed, with regular side effect monitoring. We demonstrate therapeutic potential of CNF‐DBS in chronic SCI with an acceptable risk profile in the 2 patients, along with insights resulting in protocol optimizations for future patients.

## Methods

### Study Design

In this work, we present results from the first 2 participants (P1 and P2) of a clinical pilot trial investigating feasibility, side effects, and therapeutic potential of CNF‐DBS to support neurorehabilitative training and improve gait after severe incomplete SCI (clinicaltrials.gov, NCT03053791). The detailed protocol with defined end points and eligibility criteria was published previously^23^ and follows the Standard Protocol Items: Recommendations for Interventional Trials (SPIRIT) guidelines.^24^ After baseline examinations, a DBS system was stereotactically implanted unilaterally into the CNF of the less severely affected side based on clinical examination of residual motor function (the left side in both patients). Unilateral implantation on the less severely affected side was chosen due to dependence of CNF‐DBS‐initiated stepping on ipsilaterally spared reticulospinal fibers,^15^, ^25^ bilateral reticulospinal action,^26^ midline‐crossing reticulospinal projections post‐SCI,^27^, ^28^ preclinical evidence,^13^, ^16^ and lower invasiveness. Subsequently, patients underwent CNF‐DBS‐assisted training with regular follow‐up assessments performed without (off‐condition, DBS_OFF) and with (on‐condition, DBS_ON) stimulation for 6 months. The primary outcome was the change in the 6MWT distance after 6 months versus baseline (≥ 30% improvement = success). Secondary end point measures assessed motor, autonomic function, and patient wellbeing. Power analysis for sample size definition for each task was not performed as data acquisition and measurement replication were limited by the patients’ capacity. End points were acquired once per timepoint and condition. Excluded data are reported in respective Methods sections. No outliers were excluded. Patient blinding to the stimulation condition was limited by perception of stimulation; the researchers were unblinded. Testing sequence remained fixed after initial definition for consistency. See the Supporting Information for further methodological details.

## Ethical Approval

Ethical approval was obtained from the Ethical Committee of the Canton of Zurich (case number BASEC 2016‐01104) and Swissmedic (10000316). Written informed consent for study participation was obtained from both participants. Publication of the 2 patients’ data was specifically approved by the Ethical Committee of the Canton of Zurich. We complied with all relevant ethical regulations.

### Stereotactic Electrode and Implantable Pulse Generator Implantation

Surgical procedures (lead implantation = regional anesthesia; implantable pulse generator [IPG] implantation = general anesthesia) are detailed in the published protocol.^23^ Continuous recording of local field potentials (LFPs) during microelectrode advancement (target –10 mm to target in P1; to target +4 mm in P2; 0.5 mm steps) was followed by test stimulations with increasing amplitudes (mAs), whereas the patients performed personalized motor tests: P1 performed ankle movements supplemented by M. tibialis anterior electromyography (EMG) recording; P2 attempted any possible leg movement. DBS electrodes (model 3389‐28 cm; Medtronic, Minneapolis, MN) were implanted on the left side. P1 had a 2‐stage procedure with temporary lead externalization for 1 week (IPG: Medtronic Activa SC model 37603; extension cable: Medtronic model 37086‐60 cm). P2 received the full implant in one session. Implantation sites were reconstructed in Neuroinspire (Renishaw, Gloucestershire, UK); 3D reconstruction of lead contacts relative to surrounding nuclei/tracts was performed using Lead‐DBS.^29^ Regions of interest were reconstructed using Montreal Neurological Institute (MNI) space subcortical atlases,^30^, ^31^, ^32^ and electrode contact positions were manually verified and corrected as needed using reference templates.^33^, ^34^

### 6‐Minute Walking Test

For the primary readout,^23^ the maximal distance walked (meters [m]) on even ground within 6 minutes was recorded. Both patients were accompanied by a physiotherapist and study nurse and allowed to rest at their discretion; the number of rests (minutes, count) was documented. P1 used a wheeled forearm walker with engaged breaks and bilateral ankle orthoses as assistive devices at all assessment timepoints. P2 was tested in parallel bars (9 m length) wearing bilateral ankle and wrist orthoses at all timepoints; if the bar's end was reached within 6 minutes, timing was stopped, the patient brought back to the starting point, and timing was restarted. P1 was assessed at baseline, 2‐week, and 1‐, 3‐, and 6‐month, and an additional 4 year (3 days of 3 consecutive weeks) timepoint (the 1‐week timepoint was omitted due to IPG implantation surgery). P2 was assessed at baseline, 1‐week, 2‐week, and 1‐, 3‐, 4.5‐ (end of inpatient rehabilitation), and 6‐month timepoints.

### 10 Meter Walking Test

Walking speed (m/s) was recorded during walking on plane ground of 10 meters (P1: self‐selected and fast speed; P2: fast speed).^23^ Identical assistive devices were used as for the 6MWT. Assessments were performed at baseline, 1‐week, 2‐week, and 1‐, 3‐, and 6‐month timepoints (the 1‐week timepoint was omitted for P1 due to IPG implantation; despite multiple trials, P2 was unable to perform the 10 Meter Walking Test [10MWT] at 2‐week timepoint).

### Timed Up and Go Test

The time (in seconds) needed to rise from a chair, walk 3 meters, turn around and return to the seated position was measured.^23^, ^35^ P1 used the same assistive devices as for the 6MWT at baseline, 2‐week, and 1‐, 3‐, and 6‐month timepoints (the 1‐week timepoint was omitted due to IPG implantation). P2 was unable to perform the Timed Up and Go (TUG) test at any timepoint as he required major assistance to transition from sitting to standing.

### Kinematic Gait Analysis

Three‐dimensional kinematic analysis^23^ of the walking pattern was performed during overground locomotion supported by a walker (P1) or in parallel bars (9 m length; P2). P1 was assessed at baseline, 2‐week, and 1‐, 3‐, and 6‐month, and an additional 3‐year timepoint (the 1‐week timepoint was omitted due to IPG implantation; the 2‐week and 1‐month timepoints were not displayed as they provided no additional information). P2 underwent testing at baseline, 1 week, 2 weeks, and 1, 3, and 6 months (< 3 steps prior to implantation). Equipment of gait laboratory: 27 infrared cameras recording via Nexus 2.12 (Vicon, Oxford, UK; sampling rate of 200 hertz [Hz]), 42 reflective markers (14 mm diameter) placed on anatomic landmarks using the full‐body gait model (Plug‐in‐Gait, Vicon, UK). Kinematic data were processed in Vicon Nexus and extracted to MATLAB (R2019; Mathworks Inc., Natick, MA) for further analysis.^36^ Extracted parameters included: step length (mm); stride length (mm); stance phase (% of gait cycle in P1; seconds in P2 due to long step durations); circumduction (mm); number of steps per trial; range of motion (ROM, degrees) and trajectories (degrees) of hip/knee/ankle joints. To control for compensatory trunk/pelvis movements (eg, hip hiking) in P2, vertical displacement of the anterior iliac spine was analyzed across the gait cycle.^37^ Data are shown for both legs for P1 (asymmetric gait) and P2 (symmetric gait).

### Pre‐ and Postoperative Electrophysiological Measurements

Motor evoked potentials (MEPs) of tibialis anterior muscle and tibial nerve somatosensory evoked potentials (SSEPs) were performed at baseline and 6 months.^23^

EMG recording during gait assessment was performed using bipolar Ag‐AgCl surface EMG electrodes (Kendall H124SG; Cardinal Health, Dublin, OH) positioned bilaterally over the vastus medialis, semitendinosus, tibialis anterior, and gastrocnemius medialis muscles (no recording from hip flexors due to deep location). P2's EMG data were excluded from analysis due to weak, unreliable EMG signals.

### Deep Brain Stimulation

Postoperatively, various stimulation parameters (frequency, Hz; pulse width, μs; amplitudes, mA; voltages, V) were tested at different contacts (0–3) and polarities (monopolar and bipolar) to identify individual side effect thresholds and training parameters (Supplementary Tables S2 and S4). Initial parameters were based on preclinical (50 Hz),^13^, ^15^, ^16^ clinical (20 Hz),^33^ and intraoperative (8 Hz) data, and were adapted based on patient feedback and observed (side) effects: P1 started training with 8 Hz, 240 μs, switching to 20 Hz, 420 μs after 3 months; P2 trained with 20 Hz, 450 μs. Intensities were just below side effect threshold (V). Side effect thresholds (V) were re‐evaluated before each assessment and training. Assessments were performed without and with stimulation at each follow‐up using the latest training stimulation parameters: P1 was tested first in on‐, then off‐condition (randomized at first assessment); P2 was tested first in off‐, then on‐condition (to avoid systematic favoring of DBS_ON due to easy fatigability). Stimulation was activated/intensity increased 15 minutes before DBS_ON testing and deactivated 15 minutes before DBS_OFF testing to prevent carry‐over effects of stimulation. Stimulation strength was slightly reduced during breaks (0.1–0.2 V) for support of daily activities and turned off during sleep to grant rest. Stimulation volumes were modelled in SureTune (version 4.0.1; Medtronic, MN).

### DBS‐Assisted Training

P1 underwent inpatient rehabilitation for 6 weeks at Balgrist University Hospital and 8 weeks in an external rehabilitation center (1 month each between 1‐ and 3‐month and 3‐ and 6‐month follow‐ups), focusing on overground and treadmill locomotor training. At home, she self‐trained overground walking with DBS regularly. P2 underwent 13 weeks of inpatient rehabilitation at Balgrist University Hospital until 4.5 months after implantation, gradually increasing the amount of step training (eg, Lokomat), which was barely possible according to medical records and videos pre‐study. He then discontinued his personalized training schedule for personal reasons at home, but continued applying DBS as instructed. See Supplementary Table S3 for rehabilitation schedules.

### Data Analysis and Statistics

Given the nature of this study as a phase I/II trial and a limited sample size, the study is generally not powered for inferential statistical analysis. Thus, data are primarily presented using descriptive statistics (see figure legends). Additionally, we performed a mixed‐effect analysis to examine the effect of the fixed factors “condition” and “time” on 6 kinematic parameters (P1: stride length, stance phase, circumduction, ROM of hip/knee/ankle joint; P2: stride length, stance phase, vertical hip displacement, ROM of hip/knee/ankle joint) of the left and right leg. Mixed‐effect analysis was performed with the Geisser–Greenhouse correction to account for non‐sphericity in the dataset. For P1, the effect of “time” on gait parameters was assessed by a Kruskal‐Wallis test comparing baseline to DBS_ON‐conditions at 3 months, 6 months, and 3 years after DBS implantation. The α‐levels were adjusted for multiple testing of gait parameters (n = 6) and site (left and right leg) using Bonferroni's correction (ie, α‐level = 0.05/12). EMG time series data comparing DBS_ON‐ versus OFF‐conditions at 3 months, 6 months, and 3 years were performed using statistical nonparametric mapping (SnPM) with the open source spm1d package (version 0.4.11; https://spm1d.org/). The null hypothesis was rejected if the test statistic SnPM{t} exceeded the critical value of t* at alpha = 0.05. Data processing, analysis, and graph preparation were performed in STATA (version 14; StataCorp, College Station, TX), GraphPad (Boston, MA), Python (version 3.7), and MATLAB (R2019/R2020b/R2022b; Natick, MA). Figures were generated with Adobe Illustrator CC 2019.

## Results

### Study Participants

**FIGURE 1:**
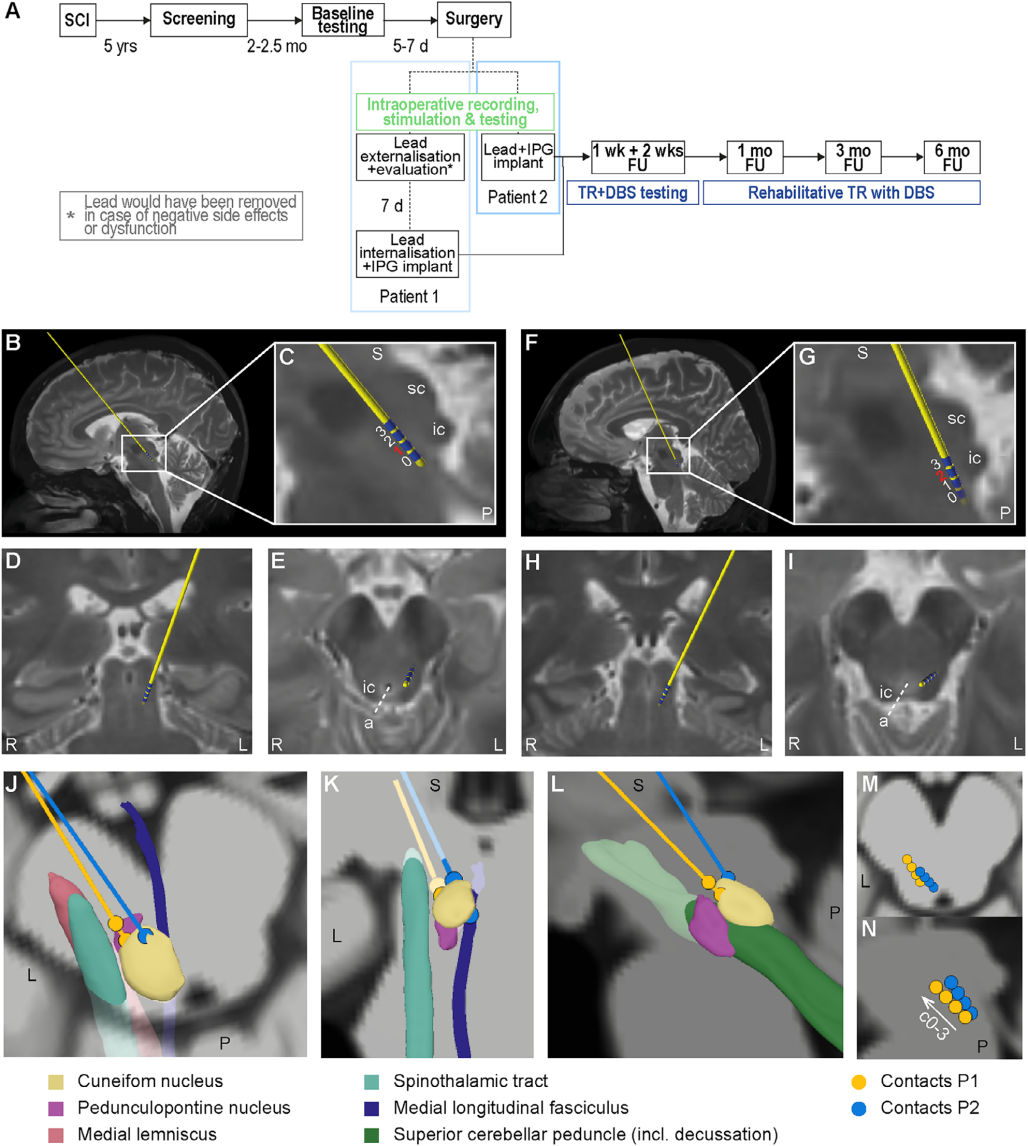
Study design and CNF targeting. (A) Study timeline of both patients. Adapted from Stieglitz and Hofer et al.^23^ (B–E) Reconstructed electrode (Medtronic 3389‐28 cm) positioning on T2‐weighted MRI in P1. (B) Sagittal projection with (C) zoom‐in of lead tip in relation to superior (sc) and inferior colliculus (ic). (D) Coronal (relation to midline) and (E) axial (relation to aqueduct [a] and ic) projection at level of contact 1. (F–I) Reconstructed electrode placement on T2‐weighted MRI in P2. (F) Sagittal projection with (G) zoom‐in of lead tip. (H) Coronal and (I) axial MRI section at level of contact 2. (B–I) Implantation trajectories varied between patients due to different parenchymal/vascular anatomy and intraoperative responses; 0–3: lead contacts; red: active contact. (J–L) 3D reconstruction (Lead DBS) of both patients’ lead contacts in relation to surrounding tracts potentially related to observed, transient side effects in (J) axial, (K) coronal, and (L) sagittal projection. Medial lemniscus: paresthesia; Spinothalamic tract: pain and temperature sensations; Medial longitudinal fasciculus: oscillopsia; Superior cerebellar peduncle: oscillopsia. (M, N) Illustration of relation between both patients' stimulation contacts in (M) axial and (N) sagittal projection. C0‐3 = contact 0–3. CNF = cuneiform nucleus; d = day(s); DBS = deep brain stimulation; FU = follow‐up; IPG = implantable pulse generator; L = left; mo = month(s); MRI = magnetic resonance imaging; P = posterior; P1 = participant 1; R = right; S = superior; SCI = spinal cord injury; TR = training; wks = weeks. [Color figure can be viewed at www.annalsofneurology.org]

Two participants were enrolled in this clinical trial (Fig 1A). Data on demographics and motor and sensory functions are shown in the Table 1 and Supplementary Table S1.

**TABLE 1:** Patient Demographic Data

	Patient 1	Patient 2
Sex	Female	Male
Age, yr	30	38
Years post‐injury	5	5
Trauma mechanism	Fall accident	Headfirst diving accident
SCI syndrome	Right‐sided, spastic Brown‐Séquard‐like syndrome	Spastic tetraparesis (right > left)
SCI severity	AIS D	AIS C
Motor level	C6	C7
Sensory level	C5	C6
Lumbar MEPs		
Left TA	Normal	Normal
Right TA	Normal	Normal
Cortical MEPs		
Left TA	↑ latency	Absent
Right TA	Absent	Absent
Tibial nerve SSEPs		
Left	Normal	Absent
Right	↑ latency	Absent
Antispasticity medication	No	No
Significant autonomic dysreflexia	No	No

AIS = American Spinal Injury Association (ASIA) impairment scale; MEPs = motor evoked potentials; SCI = spinal cord injury; SSEPs = somatosensory evoked potentials; TA = tibialis anterior muscle.

Participant P1 is a generally healthy 30‐year‐old woman with a chronic (5 years), right‐sided tetra‐spastic Brown‐Séquard‐like syndrome (American Spinal Injury Association/ASIA Impairment Scale, AIS D). Prior to inclusion, she had completed several inpatient rehabilitation programs and presented with good ability for self‐care and self‐transfers, but highly impaired mobility indoors and outdoors. Micturition and defecation were controlled voluntarily.

Participant P2 is a generally healthy 38‐year‐old man with a chronic (5 years) right‐accentuated spastic tetraplegia (AIS C). Prior to inclusion, he had completed inpatient rehabilitation after injury followed by self‐training at home since then. He presented with low‐to‐moderate ability for self‐care, no bladder and bowel control, and highly impaired ability for self‐transfers and mobility. Support was needed for micturition (Credé‐maneuvre, urinary sheath, and intermittent catheterization) and defecation (laxatives and abdominal press).

### Electrode Implantation Guided by Intraoperative Assessments

Following baseline assessments (see Fig 1A) both patients underwent left‐sided lead implantation (Fig 1B–N; Supplementary Fig S1A–F).

In P1, single microelectrode LFP recording showed theta band activation (8 Hz) in the region of interest.^33^ Test stimulations (50 Hz; 250 μs) produced right facial arm and pain with 0.3 mA at target ±2 mm, potentially from spinothalamic tract co‐stimulation. A more anterior and antero‐medial trajectory allowed up to 0.7 mA stimulation without side effects and improved ankle movement and spasticity. The anterior trajectory was chosen, and the electrode was temporarily externalized for further testing. Intraoperative M. tibialis anterior EMG (Fig S2A–D) showed higher average root mean square (RMS) values during DBS (16.2 at 0.3 mA and 18.5 at 0.5 mA) versus off‐conditions (BL1 = 11.3 and BL2 = 12.6), indicating stimulation‐induced facilitation of muscle activation. Statistical analysis confirmed significant DBS effects on intraoperative EMG activity (Friedman's 2‐way Analysis of Variance [ANOVA] by Ranks [with dependent samples] = 36.2, *p* < 0.001; post hoc comparisons: BL1 vs BL2: *p* = 1.000; BL1 vs 0.3 mA: *p* < 0.001; BL1 vs 0.5 mA: *p* < 0.001; BL2 vs 0.3 mA: *p* = 0.018; BL2 vs 0.5 mA: *p* < 0.001; 0.3 mA vs 0.5 mA: *p* = 1.000), despite limited power due to single‐patient observations. Postoperative computed tomography (CT) confirmed proper electrode positioning. No further pain occurred during a week of stimulation testing (0.1–0.7 V) for acclimatization, and P1 underwent IPG implantation.

In P2, microelectrode recording (central, anterior, posterior, and medial) noted signal alterations along the central and posterior trajectories in the region of interest. Test stimulations via the central and posterior electrode (20 Hz, 400 μs) performed ±2 mm around the target during leg movements caused minimal, intensity‐dependent oscillopsia; the central lead slightly improved leg motion (subjectively), which was chosen for implantation. CT confirmed correct electrode positioning and the IPG was implanted. After initial uneventful recovery, P2 developed transient non‐directional diplopia and anisocoria (right > left) without headache, altered consciousness, or impaired pupillary/oculomotor function. Emergency CT ruled out complications, and symptoms resolved completely and spontaneously within hours.

### Parameter Selection and CNF‐DBS‐Assisted Rehabilitative Training

Post‐implantation, patients’ side effect and motor response profiles were assessed by applying a variety of stimulation settings. Being most reliable, stimulation intensities inducing side effects (V) were defined as reference for training parameter selection.

P1's most commonly reported side effect was right‐eye oscillopsia (see Supplementary Table S2). During probatory treadmill training with body weight support, stimulation (50 and 8 Hz) yielded positive effects (Supplementary Fig S2E). CNF‐DBS also enhanced MOTOmed (RECK‐Technik GmbH & Co. KG, Betzenweiler, Germany; Supplementary Fig S2F) training intensity and peak power. Although no large‐scale on–off motor effects were observed, P1 reported facilitated walking and feeling of improved endurance when stimulated via contact 1 with 8 and 20 Hz and ≥ 120 μs pulse widths (see Supplementary Table S2). Monopolar stimulations were better tolerated than bipolar. P1 thus trained with monopolar DBS (contact 1) at 8 Hz, 240 μs, sub‐side effect threshold (≈0.9–1.1 V). Due to a higher side effect threshold with similar motor effects that emerged during parameter re‐evaluation after 3 months, stimulation parameters were changed to 20 Hz, 420 μs thereafter. P1 performed trainings as planned (see Supplementary Table S3) during the study period. She continued annual inpatient rehabilitation and regular self‐training with DBS support beyond study participation (20 Hz, 420 μs, new: contact 0), without additional side effects or threshold shifts.

P2 reported oscillopsia (8, 20 Hz) and double/blurred vision (50 Hz) at supra‐threshold stimulations postoperatively (see Supplementary Table S4). Whereas 50 Hz stimulations tended to hinder walking due to increased spasticity, monopolar stimulation with 20 Hz (contact 2) improved leg movements. He thus trained with monopolar DBS at contact 2, 20 Hz, 450 μs, at subthreshold intensities (0.7–1.3 V), as outlined in Supplementary Table S3; at home, he discontinued regular training for personal reasons but continued to apply DBS as instructed.

The 3D reconstruction of lead positioning in relation to surrounding structures (see Fig 1J–N) shows a slightly more ventral and more lateral positioning of the contact used for stimulation during the study period in P1 (contact 1) compared to P2 (contact 2).

### Walking Distance and Time after CNF‐DBS‐Assisted Training

Motor, sensory, functional, and spasticity scores for both patients are depicted in Supplementary Tables S1 and S5. Both patients presented with a stable pre‐stimulation walking distance: walking distances covered during the 6MWT differed by 4 m between screening (141 m) and baseline (137 m) in P1 and by 1.1 m between screening (3.7 m) and baseline (4.8 m) in P2. Gait assessments, conducted with consistent assistive devices (see the Methods section), were performed without and with stimulation. No carry‐over effects were observed when switching between DBS_ON and DBS_OFF.

**FIGURE 2:**
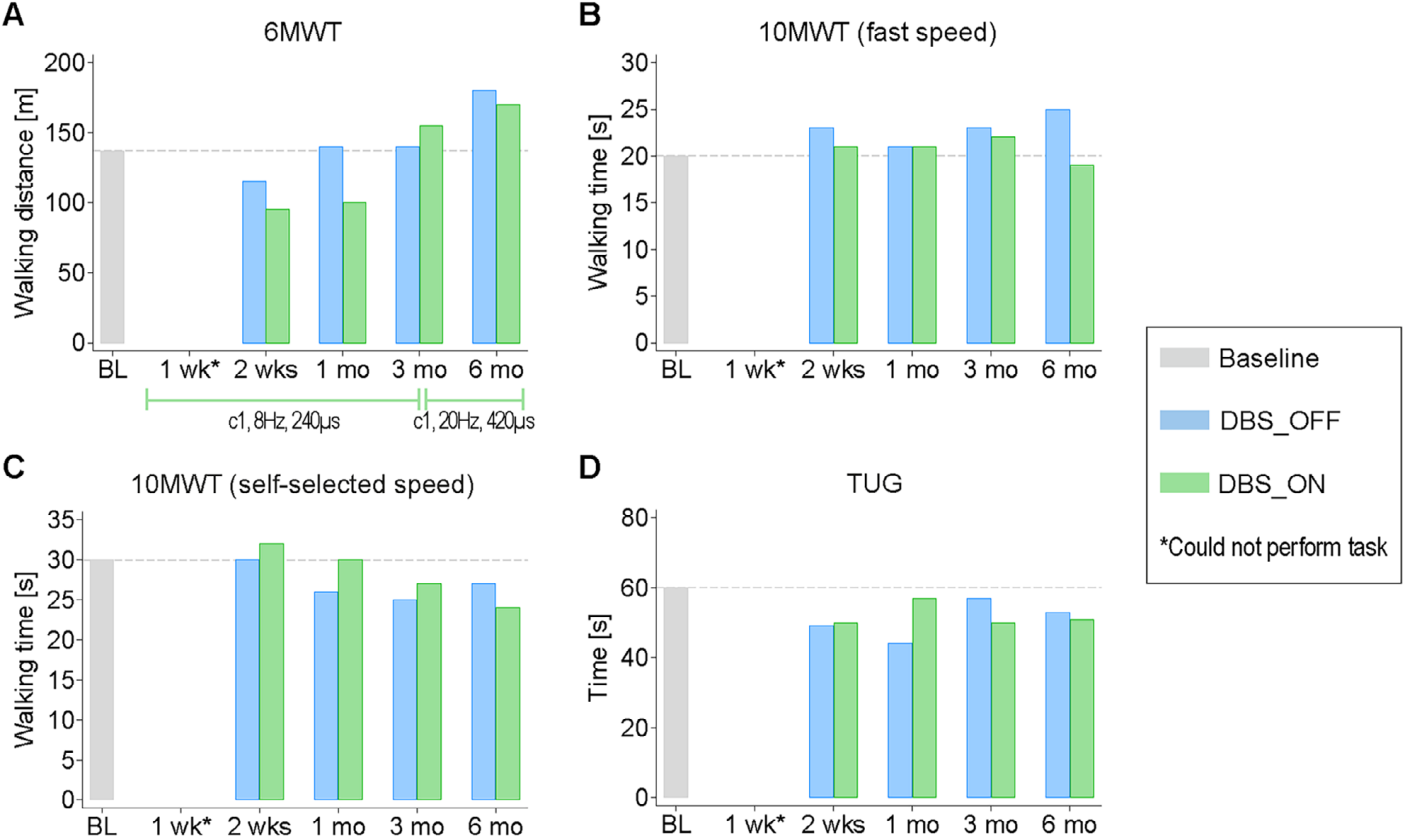
Walking distance and time of P1. (A) Walking distance (m) covered during the 6MWT at baseline and follow‐ups without and with DBS; 30% improvement at 6 months = primary study end point reached in off‐condition. Walking time (seconds) during the 10MWT at (B) fast speed and (C) self‐selected speed at baseline and follow‐ups without and with DBS. (D) Time (seconds) needed to perform TUG at baseline and follow‐ups without and with DBS. (A) Green horizontal lines = stimulation parameters used during training per time interval; applicable to (A–D) with c = active lead contact, Hz = frequency, μs = pulse width. Grey dashed horizontal line in (A–D) = baseline value. (A–D) Data represent absolute value of single patient observation per timepoint and condition (single measures). X‐axis in (A–D) = assessment timepoint. 6MWT = 6‐Min Walking Test; 10MWT = 10‐Meter Walking Test; BL = baseline; DBS = deep brain stimulation; DBS_OFF = without stimulation; DBS_ON = with stimulation; m = meters; mo = month(s); P1 = participant 1; s = seconds; TUG = Time‐Up and Go‐Test; wk(s) = week(s). [Color figure can be viewed at www.annalsofneurology.org]

After 6 months of DBS‐assisted training, P1's left hip and knee muscle tone normalized (see Supplementary Table S5), with a subjective reduction of spasticity. Walking distance during the 6MWT improved in both DBS_ON and DBS_OFF (see Fig 2A), exceeding the 30% improvement cutoff for successful study participation (DBS_OFF) and the minimal detectable change for clinically meaningful changes in patients with SCI of 22%^38^ (both conditions). A 4‐second rest was required with DBS at the 2‐week follow‐up. At 1 month, gait timing parameters favored DBS_OFF according to sensor‐based gait analysis (Supplementary Fig S3), suggesting a transient DBS‐induced disruption of the established walking pattern. With adaptation to stimulation, faster and larger steps emerged over time (see Supplementary Fig S3A–C) in both conditions, stepping speed variability improved (see Supplementary Fig. S3D), and single support phase increased in the right leg (see Supplementary Fig S3E), likely aiding left‐leg swing (see Supplementary Fig S3C). Endurance markedly improved over time (see Supplementary Fig S3F) in both conditions. No consistent on–off‐effects emerged in regular 6MWT, but a reproducibly larger walking distance with DBS was detected after 4 years of continued DBS (20 Hz, 420 μs, contact 0 during preceding 1.5 years; see Supplementary Fig S3G). The 10MWT and TUG performance also improved slightly in both conditions (see Supplementary Fig 2B–D).

**FIGURE 3:**
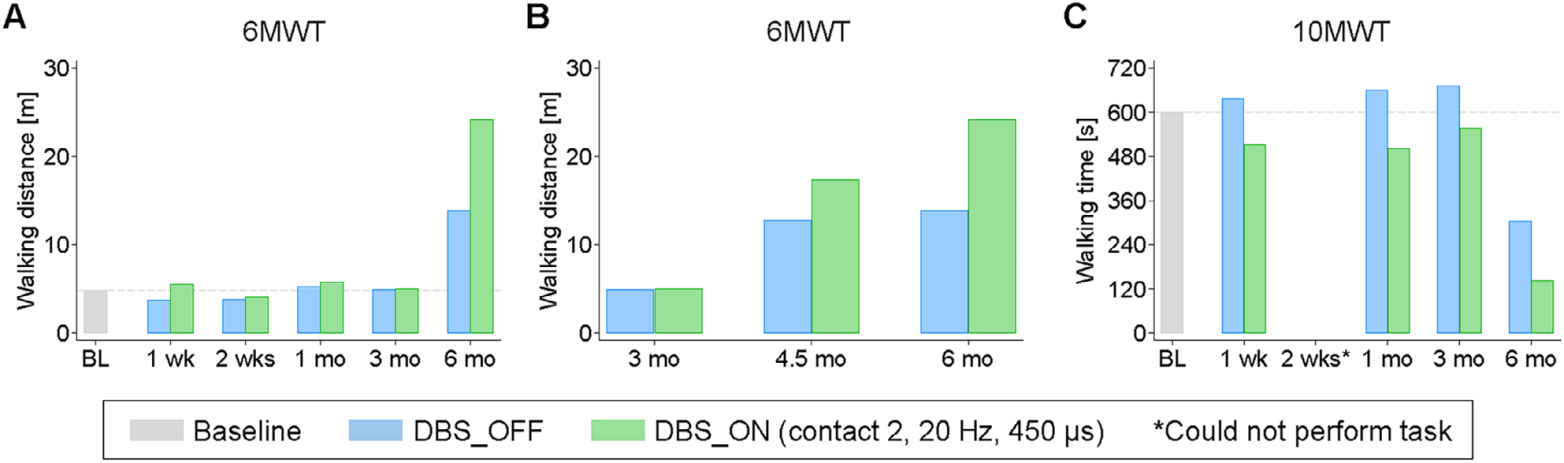
Walking distance and time of P2. (A) Walking distance (m) covered during the 6MWT at baseline and follow‐ups without and with DBS; 30% improvement at 6 months = primary study end point reached in both conditions. (B) Walking distance during (3 months), at end (4.5 months), and after (6 months) inpatient rehabilitation without and with DBS; performance‐enhancing effect of CNF‐DBS persisted beyond training discontinuation (6 vs 4.5 months; CNF‐DBS monotherapy). (C) Walking time (seconds) required during the 10MWT at baseline and follow‐ups without and with stimulation. Grey dashed horizontal line in (A) and (C) = baseline value. (A–C) Data represent absolute value of single patient observation per timepoint and condition (single measures). X‐axis in (A–C) = assessment timepoint. 6MWT = 6‐Min Walking Test; 10MWT = 10‐Meter Walking Test; BL = baseline; CNF = cuneiform nucleus; DBS = deep brain stimulation; DBS_OFF = without stimulation; DBS_ON = with stimulation; Hz = frequency; m = meters; mo = month(s); P2 = participant 2; μs = pulse width; s = seconds; wk(s) = week(s). [Color figure can be viewed at www.annalsofneurology.org]

P2's 6MWT performance improved markedly in both off‐ and on‐conditions after 6 months of CNF‐DBS‐assisted training, clearly favoring stimulation (Fig 3A,B). Walking improvement exceeded the 30% cutoff (primary end point) in both conditions. P2 required one rest at baseline (19 seconds), 1 week (10 seconds), and 6 months (50 seconds) without stimulation, but did not have to pause with stimulation at any timepoint. Following irregular self‐training with continued DBS at home (see Supplementary Table S3; months 2 to 3), walking distance improved clearly in both conditions during inpatient rehabilitation (3–4.5 months; see Fig 3B). After discharge (4.5 months), he discontinued training but continued to consequently apply CNF‐DBS daily. Off‐condition walking distance remained stable (6 vs 4.5 months), whereas walking with DBS improved further. This DBS‐induced gain beyond the training phase (4.5–6 months) exceeded the 22% threshold for a clinically relevant change in incomplete SCI,^38^ suggesting a direct stimulation effect rather than training‐induced effect. P2's 10MWT performance improved similarly, with reduced walking time in both conditions at 6 months versus baseline (Fig 3C). Clear on–off‐effects favored CNF‐DBS.

### Effect of CNF‐DBS‐Assisted Training on Walking Pattern

**FIGURE 4:**
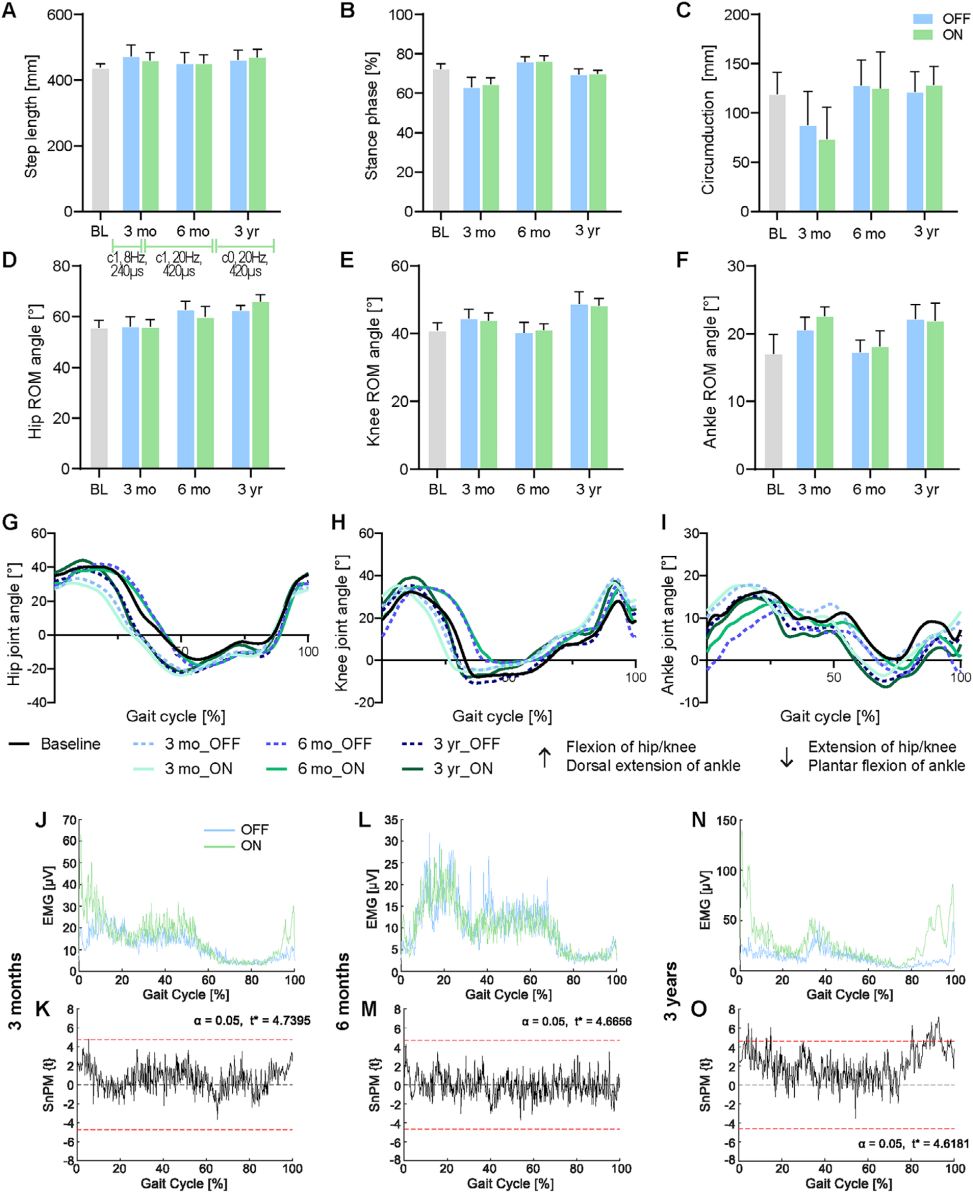
Right‐leg kinematic parameters during overground locomotion of P1. (A) Step length (mm), (B) stance phase proportion of gait cycle (%), and (C) circumduction (mm) without and with stimulation. Range of motion (ROM; degrees) of (D) hip, (E) knee, and (F) ankle joint without and with stimulation. Trajectories of (G) hip, (H) knee, and (I) ankle joint at baseline and without (OFF) and with (ON) DBS after 3 months, 6 months, and 3 years. (J‐O) M. vastus medialis electromyography (EMG; μV) of right leg (J) 3 months, (L) 6 months, and (N) 3 years after implantation with (K, M, O) SnPM (Statistical non‐Parametric Mapping; *t* = *t*‐test) analysis comparing DBS_ON vs. DBS_OFF per timepoint. (A) Green horizontal lines = stimulation parameters used during training per period; applicable to (A–O) with c = active lead contact, Hz = frequency, μs = pulse width. (A–F) Data are presented as mean + SD. X‐axis in (A‐F) = assessment timepoint. (A–O) Data shown for more severely affected right leg; data on left leg shown in Fig S4. (G–I) Dotted lines = DBS_OFF; solid lines = DBS_ON. BL = baseline; mm = millimeters; mo = months; OFF = without stimulation; ON = with stimulation; P1 = participant 1; yr = years. [Color figure can be viewed at www.annalsofneurology.org]

In P1, there was one gait parameter that was significantly modulated by the fixed effect “condition” (ie, DBS_ON vs DBS_OFF): hip ROM was reduced under DBS_ON versus DBS_OFF (F(1.558, 102.0) = 35.45; *p* = 0.0012), although the absolute difference was low in amplitude (< 3 degrees). All other gait parameters of P1 were not significantly affected by DBS_ON versus DBS_OFF (Fig 4 and Supplementary Fig S4). In contrast, most gait parameters of the left and right leg were significantly influenced by the fixed effect “time,” indicating a change over time. Specifically, step length increased bilaterally over time (right: H (4) = 21.48; *p* = 0.0004; left: H (4) = 21.24; *p* = 0.0011; see Fig 4A and Supplementary Fig S4A). At a constant cadence and higher speed, this could explain longer step lengths and increased walking distances in the long‐run. Stance phase (right: H (4) = 62.14; *p* = 0.001; left: H (4) = 42.48; *p* = 0.0009; see Fig 4B and Supplementary Fig S4B) and circumduction in the right leg (H (4) = 26.57; *p* = 0.0008; see Fig 4C) decreased at 3 months but recovered afterward. In the right leg, hip ROM (H (4) = 47.82; *p* = 0.0011), knee ROM (H (4) = 46.2; *p* = 0.0012), and ankle ROM (H (4) = 40.75; *p* = 0.0008) increased over time (see Fig 4D–F), which might suggest improved leg swing. In the left leg, knee ROM also increased over time (H (4) = 30.47; *p* = 0.0012; see Supplementary Fig S4E), but hip ROM (H (4) = 35.89; *p* = 0.0011; see Supplementary Fig S4D) and ankle ROM (H (4) = 38.65; *p* = 0.0008; see Supplementary Fig S4E) decreased with CNF‐DBS‐assisted training. Joint trajectory analysis showed no notable pattern changes (Fig 4G–I and Supplementary Fig S4G–I). The SnPM analysis of leg EMG (total gait cycle; 11–33 cycles per timepoint/condition) comparing DBS_OFF versus DBS_ON at each timepoint (see Fig 4J–O and Supplementary Fig S4J–O) revealed significantly increased EMG activity with DBS in the right M. vastus medialis around heel strike 3 years post‐implantation (see Fig 4N, O and Supplementary Fig S4N, O), possibly reflecting DBS‐induced facilitation of leg swing. However, functional relevance remains unclear due to limited kinematic changes detected between DBS_ON versus DBS_OFF.

**FIGURE 5:**
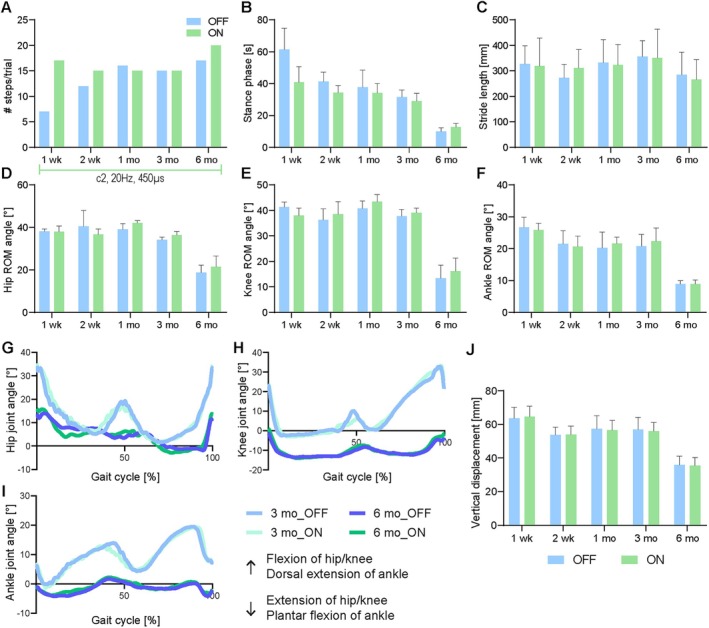
Right‐leg kinematic parameters during overground locomotion of P2. (A) Number of steps per trial (4 m distance) after implantation without and with stimulation (< 3 steps before implantation; > 15 steps at 6 months). (B) Stance phase duration (s) and (C) stride length (mm) without and with stimulation. ROM (degrees) of (D) hip, (E) knee, and (F) ankle joint without and with stimulation. (G) Hip, (H) knee, and (I) ankle joint trajectories without and with DBS at the 3‐month and 6‐month timepoints. (J) Vertical right anterior iliac spine displacement (compensatory movements by trunk/pelvis) without and with DBS. (A–F) No pre‐implantation baseline values depicted as number of steps was insufficient for kinematic analysis (< 3) prior to implantation. (A) Green horizontal line = stimulation parameters used during training; applicable to (A–J) with c = active lead contact, Hz = frequency, μs = pulse width. (A–F, J) Data are presented as mean + SD. X‐axis in (A–F) and (J) = assessment timepoint. (A–J) Data shown for more severely affected right leg; data on left leg shown in Supplementary Figure S5. DBS = deep brain stimulation; m = meters; mm = millimeters; mo = month(s); OFF = without DBS; ON = with DBS; P2 = participant 2; ROM = range of motion; wk = week(s). [Color figure can be viewed at www.annalsofneurology.org]

In P2, the number of steps was insufficient (< 3) for kinematic analysis before implantation but improved postoperatively, possibly reflecting a lead set effect (Fig 5A and Supplementary Fig S5A). There was one gait parameter that was significantly modulated by the fixed effect “condition” (ie, DBS_ON vs DBS_OFF): stance duration (Fig 5B and Supplementary Fig S5B) of the right leg was significantly reduced under DBS_ON versus DBS_OFF (F(2.737, 34.90) = 81.68; *p* = 0.0002). All gait parameters, except stride length (Fig 5C and Supplementary Fig S5C), were significantly modulated by the fixed effect “time,” indicating adaptations of the gait pattern induced by the CNF‐DBS‐assisted training. Stance phase significantly decreased over time (right: F(2.737, 34.90) = 81.68; *p* = 0.0006; left: F(2.084, 25.00) = 82.53; *p* = 0.0008; Fig 5B and Supplementary Fig S5B), probably resulting in an improved stepping frequency over time (> 15 steps; see Fig 5A and Supplementary Fig S5A). ROM of hip (right: F(2.402, 30.63) = 101.8; *p* = 0.0007; left: F(3.044, 36.53) = 63.74; *p* = 0.0011; Fig 5D and Supplementary Fig S5D), knee (right: F(2.709, 34.53) = 198.6; *p* < 0.0001; left: F(3.294, 51.89) = 89.28; *p* = 0.001; Fig 5E and Supplementary Fig S5E) and ankle joints (right: F(2.340, 29.84) = 73.17; p = 0.0009; left: F (2.141, 25.70) = 126.2; *p* = 0.0007; Fig 5F and Supplementary Fig S5F) were bilaterally reduced over time (Fig 5D–F and Supplementary Fig S5D–F). More efficient swing initiation and antigravitational strength are indirectly reflected in sagittal joint trajectories (Fig 5G–I and Supplementary Fig S5G–I), and vertical hip displacement (Fig 5J and Supplementary Fig S5J), the latter being significantly reduced at the end of the training (right: F(3.646, 62.90) = 54.26; *p* = 0.001; left: F(2.508, 30.10) = 19.26; *p* = 0.0012). The joint trajectory peak at 40–50% of gait cycle (see Fig 5G–I and Supplementary Fig S5G–I) reflects a vaulting mechanism that increases pelvis‐to‐ground distance (see Fig 5 and Supplementary Fig S5J), aiding contralateral leg swing in the presence of limited hip/knee flexion and/or ankle dorsiflexion. Less vertical pelvic displacement at 6 versus 3 months indicates more active joint control and swing initiation with less need for compensation via hip hiking (see Fig 5G–J and Supplementary Fig S5G–J). These observations suggest that the gait pattern changed bilaterally in favor of faster step initiation after stance with reduced dependence on passive relaxation of spastic knees, decisive for significantly longer 6MWT distances over time.

### Lower Urinary Tract and Bowel Function with CNF‐DBS‐Assisted Training

Observations on the lower urinary tract and bowel function are summarized for both patients in Supplementary Table S6.

In P1, free uroflowmetry measurements showed an initial postvoid residual volume of 80 ml versus 0 ml after 6 months, respectively. Video‐urodynamic investigations at 6 months showed first detrusor overactivity at bladder volumes of 75 versus 95 ml in the DBS_OFF versus DBS_ON condition, respectively, suggesting improvement with DBS.

In P2, lower urinary tract management had to be improved for medical reasons during the study period with increased catheterization frequency and antimuscarinic agents to prevent secondary complications, limiting interpretability of Qualiveen and bladder diary. The urinary symptom profile showed a positive trend over time: the stress urinary incontinence score improved from 9 to 0; the overactive bladder score improved from 18 to 6; and the low stream score deteriorated from 4 to 9. The Neurogenic Bowel Dysfunction Score improved from severe (15) to moderate (11) dysfunction over time. Video‐urodynamic investigations showed improvement comparing DBS_OFF versus DBS_ON: the volume at first detrusor overactivity increased from 300 to 390 ml with DBS.

### Side Effects of CNF‐DBS Assisted Gait Training

Patients were monitored for side effects during threshold evaluation and DBS‐assisted training, and changes in general wellbeing not directly associated with DBS‐assisted training throughout the study.

**FIGURE 6:**
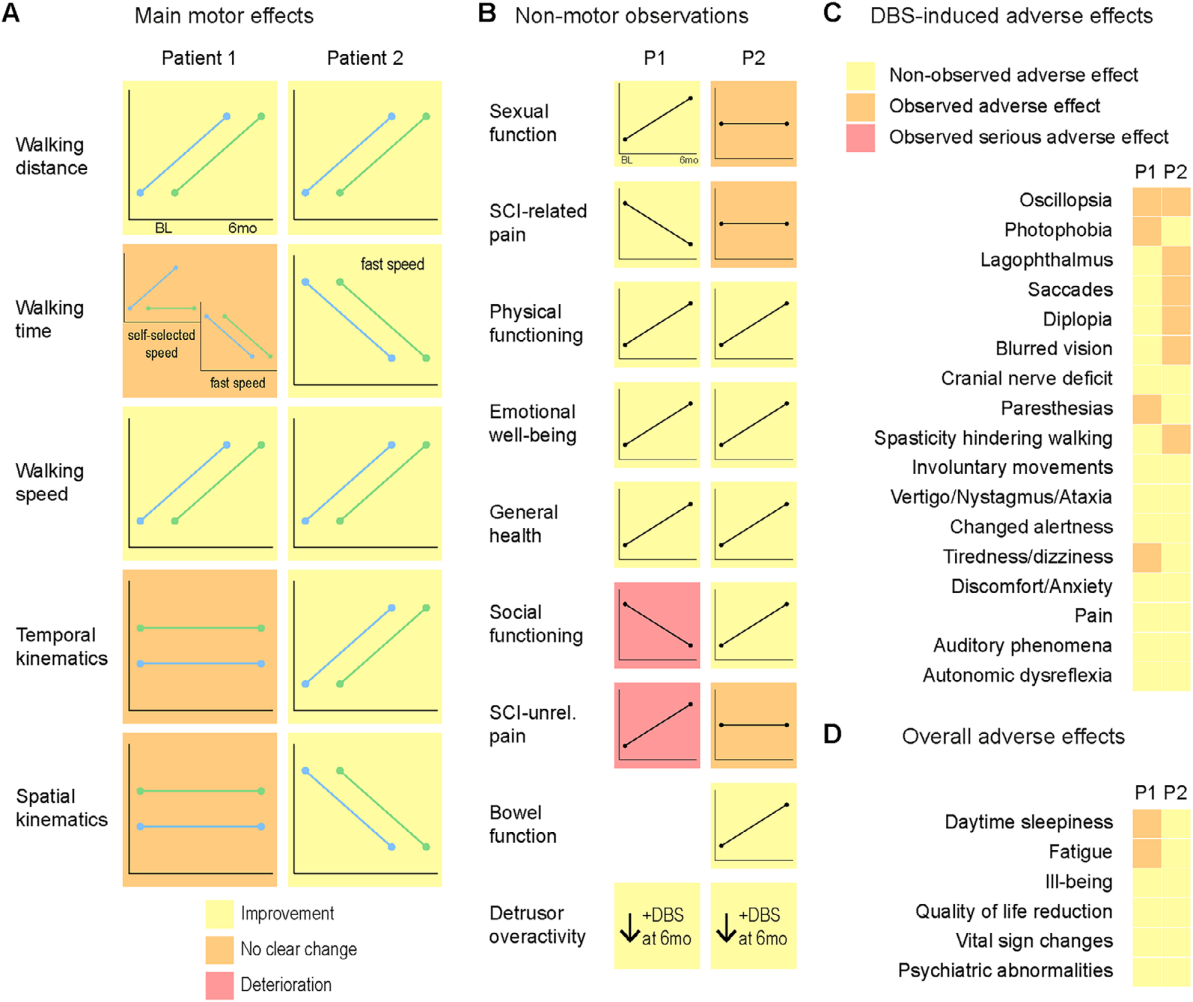
Summary of motor and non‐motor observations of P1 and P2. (A) Overview of motor effects yielded by CNF‐DBS‐assisted training over 6 months. (B) Non‐motor observations throughout 6 months of CNF‐DBS‐assisted training. (C) DBS‐induced adverse effects (training‐intensity DBS that required adjustment or supra‐threshold intensity DBS). Paresthesias reported by P1: once transient cold sensation in face; once transient tingling in right hand. All side effects were completely reversed upon reduction of stimulation intensity. (D) Overall adverse effects of CNF‐DBS‐assisted training over time (observed during off‐condition or low‐intensity stimulation used during breaks; includes data obtained from questionnaires depicted in Supplementary Table S7). 6mo = 6‐months timepoint; BL = baseline; CNF = cuneiform nucleus; +DBS = with deep brain stimulation; P1 = participant 1; P2 = participant 2. (A, B) Plots are schematic and do not provide any information about the magnitude of effects. [Color figure can be viewed at www.annalsofneurology.org]

The most commonly reported side effect at supra‐threshold intensities was oscillopsia in both patients (see Supplementary Tables S2 and S4), which completely regressed upon reduction of stimulation intensity. No stimulation‐induced neurological deficits or serious adverse events occurred at any intensity or any timepoint. With therapeutic CNF‐DBS applied during training, we observed no signs of anxiety or discomfort (periaqueductal grey, dorso‐medial); changes of alertness (locus coeruleus, caudo‐medial); vertigo, nystagmus, or ataxia (superior cerebellar peduncle incl. decussation, dorso‐medial); jaw or palatal movements (mesencephalic trigeminal nucleus/central tegmental tract, medial); paresthesia (medial lemniscus, ventro‐lateral); pain and temperature sensations (spinothalamic tract, dorso‐lateral); auditory phenomena (lateral lemniscus, lateral).^39^, ^40^ Over the study course, fatigue and daytime sleepiness increased in P1 (Supplementary Table S7) unrelated to training‐intensity DBS. Figure 6 summarizes the main motor results (see Fig 6A), non‐motor observations (see Fig 6B), training‐intensity or supra‐threshold DBS‐induced (see Fig 6C) and overall (not directly training‐intensity or supra‐threshold DBS related; see Fig 6D) adverse effects detected in this study.

## Discussion

Here, we present the first 2 patients with incomplete tetraplegia who received CNF‐DBS. Interpreted within the context of a pilot study, the reported data suggest that CNF‐DBS combined with rehabilitative training is well tolerated and can improve ambulation.

Given limited clinical data, we established a comprehensive protocol with a broad testing battery to assess side and therapeutic effects of CNF‐DBS. Both patients tolerated surgeries, stimulations, and DBS‐assisted training without serious adverse events. Supra‐threshold intensities induced intensity‐dependent oscillopsia, likely due to current spread to the trochlear nerve, or alternatively the medial longitudinal fasciculus or superior cerebellar peduncle. Symptoms resolved with reduced stimulation strength. This observation aligns with previous reports on CNF‐DBS in patients with Parkinson's disease.^33^, ^41^ Both patients exhibited narrow therapeutic windows, however, particularly P2 responded to very low intensities.

P1, a “slow walker” with a stable pre‐stimulation walking distance, reached the primary end point in the off‐condition. She showed an asymmetric Brown‐Séquard‐like walking pattern with complex compensatory use of the stronger leg, complicating data interpretation. Despite limited functional gains within the study period due to this asymmetric gait hindering sufficient bilateral leg training, she gained endurance and walking distance in the long‐run, which she also reported as a subjective positive effect. Statistical analysis, even though of limited power, supported the development of an improved gait pattern in the long‐run, with limited direct on–off effects.

P2, a “standing non‐walker” at inclusion with a stable pre‐stimulation walking distance, showed a symmetric but severely impaired gait and was forced to train both legs equally. He reached the primary end point with (24.15 m) and without (13.85 m) DBS, marking a 400% improvement from baseline (≈5 percentage points compared to a healthy control person with average walking speed of 4–6 km/h^42^). Without stimulation, he required a 50‐second break; with DBS, he walked continuously. Assuming constant speed, he would have walked 20.8 m with a 50‐second break with DBS versus 13.85 m without DBS. This indicates improved endurance and speed. P2 appeared to adopt a new gait strategy during the study period with more active swing initiation and step execution (ie, higher step frequency and reduced stance duration), and reduced compensatory movements (ie, less hip hiking). This is supported by statistical analysis, although the interpretability of these results is restricted by the low power. Less physiotherapeutic support required for walking and sustained improvement without stimulation suggest lasting adaptation of motor function. Although the specific contributions of training, DBS, or their combination remain unclear, continued functional gains with DBS monotherapy after training cessation suggest a contributing DBS‐related effect. Subjectively, P2 reported better lower body control with stimulation.

Besides varying lesion patterns, different lead positioning may explain the milder effects observed in P1. In P1, contacts 2 and 3 were located too superior whereas contact 1, located at the level of the inferior colliculus, yielded a satisfying risk–benefit profile and was thus chosen for stimulation during the study period. In P2, contacts 0 and 1 were positioned too medial, and contact 2 was chosen for stimulation based on its location and risk–benefit‐profile. Interpatient comparison of active contacts shows a slightly more ventral and more lateral location in P1 versus P2. Inconsistent on–off‐effects on walking distance and time were observed in P1 during the study period, possibly due to co‐stimulation of the PPN.^34^, ^43^ Switching to the more dorso‐medially located contact 0 in P1, located slightly more posterior than contact 2 of P2, for continued CNF‐DBS‐assisted training after the regular study period produced clearer on–off‐effects on walking distances, favoring DBS in the long‐run (4 years post‐implantation). This might propose that stimulation contacts should target the central to posterior part of the CNF in terms of posteriority, which is also suggested by preclinical and first clinical literature.^6^, ^33^, ^44^, ^45^, ^46^, ^47^ In terms of laterality, a central positioning of the stimulating contact in the left/right half of midbrain seems a proper starting position for intraoperative testing, and rostro‐caudally, the stimulating contact should be leveled with the inferior colliculus. However, targeting remains difficult, and directional electrodes should be used for future patients to better direct stimulation.^33^, ^41^

Lower urinary tract dysfunction also significantly affects life after SCI.^48^ At 6 months, both patients showed delayed detrusor overactivity during stimulation in video‐urodynamic assessments, suggesting a direct DBS‐induced effect.^49^ Longitudinal conclusions are limited by ≥ 6 month inter‐assessment intervals and bladder management optimization in P2. Anecdotally, P2 reported improved bladder sensation, micturition frequency, and control with DBS, effects that outlasted stimulation. Beneficial effects on autonomic function with locomotor training were reported clinically^50^ and with CNF‐DBS‐assisted training preclinically.^13^ Interactions between MLR and pontine micturition center (Barrington's nucleus) might be explanatory anatomical correlates.^6^, ^51^, ^52^, ^53^

Our findings show that the CNF‐reticulospinal system can be modulated in patients with chronic incomplete SCI. No serious side effects and no stimulation intensity‐dependent side effects that were not reversible upon reduction of stimulation strength were observed in the 2 included patients, who generally presented without autonomic dysreflexia. However, as CNF stimulation can affect the cardiovascular system,^45^, ^47^ the autonomic side effect profile should be specifically assessed in case vulnerable patients with autonomic dysreflexia undergo CNF‐DBS in the future. Given generally limited on–off‐effects across all parameters, CNF‐DBS appears to support functional gait development over time in combination with rehabilitative training, rather than directly imposing a new gait pattern. Besides the reticulospinal tract, other motor pathways might contribute to these changes. In such patients with severely impaired gait, functional gait relies on compensatory strategies, making adaptive gait changes clinically meaningful. Given our study design demanding constant assistive devices and walking‐focused rehabilitation, WISCI II and SCIM could not capture such subtle improvements. Nevertheless, our findings support that (asymmetric) gait can be modulated by stimulation^54^ and training^55^ even years after injury. Based on the minimal changes observed in P1, we propose 2 strategies for future patients with similar lesions: (1) early intervention before compensatory patterns solidify, and/or (2) pre‐DBS training targeting weaker leg use. Delayed improvements seen across various assessments in both patients may indicate the need for long‐term stimulation to maximize therapeutic benefit. Although the ideal stimulation paradigm for CNF‐DBS in SCI is unknown, the one applied in this study was designed to use CNF‐DBS as enabler or enhancer of movement and to strengthen functionally meaningful neural circuitries through spike‐timing dependent plasticity. Continuous stimulation is a potential alternative to be tested in the future, and might be suitable for patients earlier after injury to promote reticulospinal plasticity.

Alternatively to DBS, epidural and transcutaneous spinal cord stimulation also show promise.^9^, ^10^, ^11^ They supposedly enhance spinal locomotor circuit activity via sublesional somatosensory dorsal root fiber stimulation.^56^, ^57^ In contrast, CNF stimulation triggers a physiological brainstem‐spinal pathway essential for walking.^3^, ^6^ Although being frequent, the anatomical prerequisite of spared reticulospinal fibers limits CNF‐DBS's therapeutic reach to anatomically incomplete SCI. Besides the CNF, the lateral hypothalamus has recently been proposed as a DBS target to improve walking after incomplete SCI.^58^ Whereas DBS of both the CNF and the lateral hypothalamus requires further testing in patients for generalizable conclusions, the CNF is the currently better‐understood target. Robust preclinical evidence across species supports the MLR–reticulospinal axis as the main pathway for locomotor control, with well characterized connectivity between MLR and reticulospinal neurons,^6^, ^25^, ^59^, ^60^ and effects of CNF stimulation have been thoroughly studied preclinically.^13^, ^15^, ^47^, ^61^ Recent publications on CNF‐DBS in patients with freezing of gait due to Parkinson's disease have demonstrated a beneficial impact on the execution of controlled stepping movements.^33^, ^41^ Our findings seem to be in line with these results, supporting the conceptual ideal that CNF stimulation promotes the ability to perform voluntary stepping over time.

Given its pilot nature, this trial has limitations. The sample size was small and the patients’ easy fatiguability innate to their severe injuries and extensive testing batteries limited repeated measures. Thus, the study is generally not powered to demonstrate statistical significance. Differing injury types led to distinct walking behaviors, further limiting generalizability of conclusions. Blinding to stimulation conditions was challenging as the patients sensed the state of stimulation even at low intensities. Vascular anatomy caused slight electrode trajectory variations as common in DBS. A further limitation of this study is the variability in the patients’ adherence to the rehabilitation protocol, which might have influenced treatment outcomes: whereas P1 followed the training schedule as instructed, her stimulation paradigm was changed after 3 months; in contrast, P2 applied the initially defined stimulation paradigm, however, he discontinued training after 4.5 months. DBS‐supported inpatient training could be well controlled in both patients. However, the transition to its integration into daily life activities and the challenges that impact patients’ self‐training abilities in the home environment require more attention in future participants.

Combining our lessons learnt, we propose selecting patients with symmetric injury patterns, standing ability, and impaired step initiation in the (sub)chronic phase to refine future implementation of CNF‐DBS.^1^, ^8^ Stratified early enrollment,^62^, ^63^ and anatomic and functional assessment of reticulospinal integrity may optimize future candidate selection. MRI‐based evaluation of reticulospinal tract preservation has been shown to correlate with recovery of mobility,^19^, ^64^ whereas residual reticulospinal function can be assessed electrophysiologically through myogenic potentials evoked by startling acoustic stimuli.^65^, ^66^ As intraoperative motor testing proved less reliable than expected, targeting will rely more on imaging and side effect monitoring. Future candidates will receive perceptive IPGs (Medtronic Percept PC B35200) and segmented leads (Medtronic Sensight B3400095) for more precise CNF modulation. The study protocol was updated and re‐approved recently. Recruitment of further patients with incomplete SCI above T10 is ongoing.

## Author Contributions

A.S.H., L.H.S., M.B., C.R.B., L.I., L.R., M.E.S., and A.Cu. contributed to the conception and design of the study; A.S.H., L.H.S., M.B., L.F., A.C., R.W., I.L., I.K., M.G., C.M., M.S., M.H., T.M.K., L.D., C.R.B., L.I., M.F.O., A.P., A.K., L.R., M.E.S., and A.C. contributed to the acquisition and analysis of data; A.S.H., L.H.S., L.F., A.Ca., I.L., M.G., M.H., T.M.K., A.K., M.E.S., and A.C. contributed to drafting the text or preparing the figures. [Correction added on 14 January 2026, after first online publication: Author contribution text has been revised in this version.]

## Potential Conflicts of Interest

Nothing to report.

## Supporting information


**Supplementary Data S1.** Supporting Information.

**Supplementary FIGURE S1:** Stimulation volume modeling.

**Supplementary FIGURE S2:** Intraoperative EMG and early postoperative stimulation effects in P1.

**Supplementary FIGURE S3:** Sensor‐based gait analysis of P1 during selected 6‐Minute Walking Tests.

**Supplementary FIGURE S4:** Left‐leg kinematic parameters during overground locomotion of P1.

**Supplementary FIGURE S5:** Left‐leg kinematic parameters during overground locomotion of P2.

**Supplementary Table S1.** Motor and sensory scores.

**Supplementary Table S2.** Overview of side‐effect thresholds and subjective motor observations in dependence on stimulation parameters and contacts during DBS‐testing in patient 1.

**Supplementary TABLE S3.** Training schedule during inpatient rehabilitation at Balgrist University Hospital.

**Supplementary TABLE S4.** Overview of side‐effect thresholds and subjective motor observations in dependence on stimulation parameters and contacts during DBS‐testing in patient 2.

**Supplementary TABLE S5.** Modified Ashworth Scale (MAS) of spasticity.

**Supplementary TABLE S6.** Lower urinary tract and bowel function.

**Supplementary TABLE S7.** Scoring of sexual function, sleepiness, fatigue, pain, and quality of life.

## Data Availability

The data supporting the results and conclusions are present in the paper and the Supplementary Materials, or are available from the corresponding author upon reasonable request. Previously reported computer codes or algorithms central to the conclusions are referred to in the respective Methods sections. Codes and algorithms are also available upon request.
