# Supplementary material for: Cuneiform Nucleus Stimulation Can Assist Gait Training to Promote Locomotor Recovery in Individuals With Incomplete Tetraplegia
Source: Ann Neurol. 2025 Sep 10;99(1):161–77. doi: 10.1002/ana.78026 (PMC12946608; doi:10.1002/ana.78026)
Supplement: Supplementary file 1 — Supplementary Data S1. Supporting Information. [file ANA-99-161-s004.docx]

**Supplementary Material**

Cuneiform nucleus stimulation can assist gait training to promote locomotor recovery in individuals with incomplete tetraplegia

Anna-Sophie Hofer, MD PhD^1,2^†*, Prof. Lennart H. Stieglitz, MD^1^†, Marc Bolliger, PhD^3^, Linard Filli, PhD^3,4^, Adrian Cathomen, PhD^3^, Romina Willi, PhD^3^, Irina Lerch, BSc^3^, Iris Krüsi, BSc^3^, Melina Giagiozis, MSc^3^, Christian Meyer, PhD^3^, Martin Schubert, MD^3^, Prof. Michèle Hubli, PhD^3^, Prof. Thomas M. Kessler, MD^5^, László Demkó, PhD^3^, Prof. Christian R. Baumann, MD^6^, Prof. Lukas Imbach, MD^7^, Markus F. Oertel, MD^1^, Andrea Prusse^3^, Alina Kiseleva, MSc^7^, Prof. Luca Regli, MD^1^‡, Prof. Martin E. Schwab, PhD^2^‡, Prof. Armin Curt, MD^3^‡

^1^Department of Neurosurgery, Clinical Neuroscience Center, University Hospital Zurich, Zurich, Switzerland; ^2^Institute for Regenerative Medicine, University of Zurich, Schlieren, Switzerland; ^3^Spinal Cord Injury Center, Balgrist University Hospital, University of Zurich, Zurich, Switzerland; ^4^Swiss Center for Movement Analysis (SCMA), Balgrist Campus AG, Zurich, Switzerland; ^5^Department of Neuro-Urology, Balgrist University Hospital, University of Zurich, Zurich, Switzerland; ^6^Department of Neurology, Clinical Neuroscience Center, University Hospital Zurich, Zurich, Switzerland; ^7^Swiss Epilepsy Center, Klinik Lengg, Zurich, Switzerland.

*Corresponding author: Dr. Anna-Sophie Hofer, Department of Neurosurgery, University Hospital Zurich, Switzerland. E-mail: [anna-sophie.favre-hofer@paraplegie.ch](mailto:anna-sophie.favre-hofer@paraplegie.ch).

†These authors share first-authorship.

‡These authors share senior authorship.

**Methods**

***ASIA Impairment Scale (AIS) assessment***

The American Spinal Injury Association (ASIA) impairment scale (AIS)^1^ was used to assess the neurological status at baseline and all follow-ups by a certified neurologist: A = Functionally complete SCI; B = Functionally sensory incomplete, motor complete SCI; C = Functionally motor incomplete (± some preservation of sensory function); D = Motor incomplete with ≥50% of key muscle function with muscle grade ≥3; E = Intact motor and sensory function. As part of AIS, the lower extremity motor score (LEMS) assessed motor function from 0 (no motor function) to 5 (full motor function) across the 5 lower extremity muscle groups (maximum 50 points, 25 per side); the lower extremity sensory score (0 – absent, 1 – reduced, 2 – normal) assessed sensory function for pin prick and light touch for dermatomes L1 to S4-5 (maximum 18 points per leg).

***Intraoperative electrophysiological measurements***

Intraoperative stimulation and testing were accompanied by wireless surface EMG recording (Noraxon Ultium System, Scottsdale, AZ, USA). During alternating maximal ankle plantar- and dorsiflexion, EMG of M. tibialis anterior was recorded in P1, guided by an auditory cue at 1.6 Hz; in P2, EMG of all major leg muscles was recorded during trials of unilateral and bilateral leg movements with and without facilitation to detect EMG changes. Data is shown for P1 (left M. tibialis anterior; fig. S2 A-D): each measurement lasted ≈17.5 s (cut according to actual EMG activity), sampled at 2000 Hz, bandpass-filtered (4th order Butterworth, recursive) with cut-off frequencies of 10 Hz and 500 Hz. EMG of left M. tibialis anterior was statistically analyzed for potential intraoperative on-off-effects. EMG data were cut according to maximal plantarflexion per active movement cycle and analyzed using Friedman's two-way Analysis of Variance by Ranks (dependent samples) comparing four different conditions (DBS_OFF [BL1], DBS_OFF [BL2], DBS_ON [0.3mA], and DBS_ON [0.5mA]), with pairwise post-hoc comparisons with Bonferroni correction for multiple comparisons. Intraoperative EMG data of P2 had to be excluded from analysis as no reliable temporal correlation between repetitive voluntary movement, stimulation and EMG signal was possible due to generally weak muscle activity and strong exhaustion over time.

***Sensor-based gait analysis***

At the time of inclusion of P1, sensor-based (ZurichMove sensors, www.zurichmove.com) gait analysis^2,3^ of 6MWT performance was under development to supplement kinematic analysis. During selected 6MWT (1-, 2-, 3-months; 4-years), wireless sensors were mounted to P1’s wrists and ankles for pilot-testing. Parameters were extracted by applying a gait detection algorithm^2^ in MATLAB (R2022b; Mathworks Inc., Natick, MA, USA) with personalized thresholds to detect steps and gait events according to the patients’ walking profile. Sensor data analysis was performed in Python (version 3.7). Extracted parameters included walking speed (m/s), cadence (steps/min), swing phase (%) and single/double support phase (%) of total gait cycle, smoothness, and endurance/fatigue during activity. Smoothness^4^ (consistency and continuity of movement) was quantified by the frequency spectrum of the sagittal angular velocity using the spectral arc method (values closer to zero suggest smoother movements compared to more negative values; lower smoothness indicates higher variations). Endurance/fatigue reflects the relative change of walking speed during the 6MWT (negative value = speed decrease/fatigue; positive value = increase/endurance). The data of 6MWTs performed on 3 days of 3 consecutive weeks 4 years after implantation was combined and down-sampled by retaining every third data point for comparison to earlier timepoints.

***Spinal Cord Independence Measure (SCIM III)***

The SCIM III^1^ was performed to assess overall functional ability at baseline and all follow-ups. It assesses 19 tasks across 3 subscales: self-care, respiration & sphincter management, mobility. The combined scores on all tasks yield an overall score from 0 to 100 (higher scores reflect greater functional ability). Interpretability is limited as SCIM is susceptible to ceiling and ground effects in the chronic stage in tetraplegic patients.^5^

***Walking Index for Spinal Cord Injury (WISCI II)***

The WISCI II^1^ was performed at baseline and all follow-ups to assess the amount of physical assistance and devices required for walking on an ordinal scale. Scores range from 0-20, with 0 indicating inability to walk with maximum help and 20 indicating independent walking for 10 m without any assistance or assisting devices. Interpretability is limited as WISCI only detects large changes, rehabilitation focused primarily on walking rather than activities of daily living, and assistive devices were kept constant on purpose for consistent testing over time.

***Modified Ashworth Scale (MAS)***

The MAS^1^ was performed to assess muscle spasticity at baseline and 1-week, 1-, 3-, 6-months follow-ups. Scores range from 0 (no increase in tone) to 4 (affected part in rigid flexion/extension).

***Assessment of lower urinary tract function***

Autonomic function was assessed due to known projections between the MLR and the pontine micturition center,^6^ and preclinical and clinical evidence on improved bladder function with CNF-DBS-assisted training^7^ and regular locomotor training,^8^ respectively. Lower urinary tract function was evaluated according to study protocol.^1^ The Qualiveen was employed at screening and 6 months to assess patients’ self-judgement of lower urinary tract function. It consists of 30 items across 4 domains: inconvenience/bother with limitations, restrictions/frequency of limitations, fears, feelings/impact on daily life. Each item is scored from 0 (no impact) to 4 (high adverse impact), resulting in an average score per domain and a total average score (lower scores indicate better quality of life). In addition, both patients kept a three-day bladder diary at screening and 6-months. P1 additionally underwent free uroflowmetry (not applicable for P2), and both underwent video-urodynamic investigations at screening (2 runs, expressed as mean) and 6 months (1 run DBS_OFF, 1 run DBS_ON). In P2, the Urinary Symptom Profile Questionnaire^9^ and Neurogenic Bowel Dysfunction score^10^ were performed at screening and 6 months (not applicable for P1). The Urinary Symptom Profile Questionnaire consists of 13 items in 3 dimensions: stress urinary incontinence (scored 0-9), overactive bladder (scored 0-21), low stream (scored 0-9), with higher scores indicating worse symptoms. The Neurogenic Bowel Dysfunction score is a 39-item questionnaire assessing anal incontinence, constipation, obstructed defecation, and impact on quality of life, resulting in a total score of 0-47 (each item has weighted response base; 0-6 = very minor, 7-9 = minor, 10-13 = moderate, ≥14 = severe bowel dysfunction).

***Assessment of sexual function***

The Female Sexual Function Index (FSFI)^1^ to evaluate female sexual function (19 items, 6 domains – desire, arousal, lubrication, orgasm, satisfaction, pain, scored 0-6 each, resulting in a total of max. 36 points) and the International Index of Erectile Function (IIEF)^1^ to assess male erectile function, orgasmic function, sexual desire and satisfaction (15 questions scored 0/1-5, total score 1-30) were assessed at baseline and 1-, 3-, 6-months. Higher scores indicate better functioning.

***Epworth Sleepiness Scale (ESS)***

To measure general level of daytime sleepiness, the ESS^1^ was performed at baseline and 1-, 3-, 6-months. The ESS consists of a self-administered questionnaire with 8 questions, in which patients rate their tendency to doze off or fall asleep during certain activities on a scale of 0 (no chance of dozing) to 3 (high chance of dozing), resulting in a total score of 0-24 (the higher the score, the higher the patient’s average sleep propensity in daily life; total score <11 suggests absence of excessive daytime sleepiness, total score ≥11 suggests excessive daytime sleepiness).

***Fatigue Severity Scale (FSS)***

Using the FSS^1^ we evaluated the severity of fatigue symptoms at baseline and 1-, 3-, 6-months. The FSS questionnaire contains 9 statements on fatigue, patients rate their disagreement (1) or agreement (7) with the statement (rating 1-7 per statement, total score of 1-7 [9-63/9]; total score of ≥4 suggests suffering from fatigue).

***Spinal Cord Injury Pain Instrument (SCIPI)***

The SCIPI^1^ was used to evaluate pain at baseline and 1-, 3-, 6-months. The SCIPI is a 4-item yes (1) and no (0) questionnaire suggesting neuropathic pain in patients with SCI, resulting in a total score of 0-4 (0 = no neuropathic pain, 1 = possible neuropathic pain, ≥2 = probable neuropathic pain).

***Short Form Health Survey to Assess Quality of Life (SF-36)***

Assessing quality of life, we employed the SF-36^1^ to yield a profile of functional health and well-being at baseline and 1-, 3-, 6-months. The SF-36 consists of 36 general health-related questions of different categories, resulting in subscores of 0-100%, with 0 representing the greatest possible health limitation and 100 representing the absence of health restrictions.

**Supplementary Figure Legends**

**Fig. S1**. **Stimulation volume modeling.** Modeling of stimulation volume of P1 in (**A**) coronal, (**B**) sagittal, and (**C**) axial T2 MRI with used stimulation parameters of: 20 Hz, 420 µs, 1.0 V, contact 1. Modeling of stimulation volume of P2 in (**D**) coronal, (**E**) sagittal, and (**F**) axial T2 MRI with used stimulation parameters of: 20 Hz, 450 µs, 1.0 V, contact 2. (**A**-**F**) L = left; P = posterior; S = superior.**Fig S2. Intraoperative EMG and early postoperative stimulation effects in P1.** (**A**-**D**) Visual changes of intraoperative left (ipsilateral) M. tibialis anterior EMG recording during maximal ankle plantar- and dorsiflexion guided by auditory cue (length of each measurement: 17.5 s; sampling frequency: 2000 Hz; bandpass-filtered with cut-off frequencies of 10 Hz and 500 Hz) without (DBS_OFF; A, D) and with (DBS_ON; B, C) stimulation (0.3 mA, 0.5 mA). BL = baseline. (**E**) Early subjective effects of DBS (contact 1-2+) on gait observed/reported during probatory treadmill training with body weight support (FLOAT) 7 days after implantation; effects are reported in direct comparison to respective preceding condition (ON = green, OFF = blue). (**F**) Training in the MOTOmed without and with stimulation (c1 = active lead contact) with higher average power and peak power (W) with DBS (ON) compared to without (OFF). Hz = Hertz. V = Volt. MAS = Modified Ashworth Scale. ↑ = more. ↓ = less. + = improved.

**Fig. S3**. **Sensor-based gait analysis of P1 during selected 6-Minute Walking Tests.** (**A**) Walking speed (m/s), (**B**) stepping frequency (steps/min), (**C**) swing phase proportion of gait cycle (%) without and with DBS. (**D**) Smoothness, an indicator of fluctuation of walking speed (values ≈-4 = physiological; the more negative, the higher the fluctuation) and (**E**) single support phase proportion of gait cycle (%) without and with DBS. (**F**) Endurance (%) without and with stimulation (the more positive, the higher endurance and lower fatigability). (**G**) Walking distance covered in 6 minutes without and with DBS 4 years after electrode implantation on 3 separate testing days. mo = month. yr = years. d = day. DBS_OFF = without stimulation. DBS_ON = with stimulation. (A) Green horizontal line: stimulation parameters used during training per period; applicable to (A-G) with c = active lead contact, Hz = frequency, µs = pulse width. Grey horizontal bar in (C) = reference value of healthy individual at comparable walking speed. Grey dashed horizontal line in (G) = baseline value. Data are represented as mean ± SD in (A-E). (A-F) 4-year timepoint measurement was down-sampled by retaining only every third data point. X-axis in (A-G) = assessment timepoint.

**Fig. S4. Left-leg kinematic parameters during overground locomotion of P1.** (**A**) Step length (mm), (**B**) stance phase proportion of gait cycle (%), (**C**) and circumduction (mm) without and with stimulation. Range of motion (ROM; °) of (**D**) hip, (**E**) knee, and (**F**) ankle joint without and with stimulation. Trajectories of (**G**) hip joint, (**H**) knee joint, and (**I**) ankle joint at baseline and without (OFF) and with (ON) DBS after 3-months, 6-months, and 3 years. (**J**-**O**) M. vastus medialis electromyography (EMG; µV) of left leg (**J**) 3 months, (**L**) 6 months, and (**N**) 3 years after implantation with (**K**, **M**, **O**) SnPM (Statistical non-Parametric Mapping; t = t-test) analysis comparing DBS_ON vs. DBS_OFF per timepoint. (A) Green horizontal lines = stimulation parameters used during training per period; applicable to (A-O) with c = active lead contact, Hz = frequency, µs = pulse width. (A-F) Data are presented as mean + SD. BL = baseline. mo = months. yr = years. OFF = without stimulation. ON = with stimulation. X-axis in (A-F) = assessment timepoint. (A-O) Data are shown for less severely affected left leg; data on right leg shown in Fig. 4. (G-I) Dotted lines = DBS_OFF; solid lines = DBS_ON.

**Fig. S5**. **Left-leg kinematic parameters during overground locomotion of P2.** (**A**) Number of steps per trial (4 m distance) after implantation without and with stimulation (<3 steps before implantation; >15 steps at 6-months). (**B**) Stance phase duration (s) and (**C**) stride length (mm) without and with stimulation. Range of motion (°) of (**D**) hip, (**E**) knee, and (**F**) ankle joint without and with stimulation. (**G**) Hip, (**H**) knee, and (**I**) ankle joint trajectories without and with DBS at 3-months and 6-months timepoint. (**J**) Vertical right anterior iliac spine displacement (compensatory movements by trunk/pelvis) without and with DBS. (A-F) No pre-implantation baseline values depicted as number of steps was insufficient for kinematic analysis (<3) prior to implantation. (A) Green horizontal line = stimulation parameters used during training; applicable to (A-J) with c = active lead contact, Hz = frequency, µs = pulse width. (A-F, J) Data are presented as mean + SD. wk = week(s). mo = month(s). OFF = without DBS. ON = with DBS. X-axis in (A-F) and (J) = assessment timepoint. (A-J) Data shown for less severely affected left leg; data on right leg shown in Fig. 5.

**Supplementary Tables 1-7**

**References**

1. Stieglitz LH, Hofer A-S, Bolliger M, et al. Deep brain stimulation for locomotion in incomplete human spinal cord injury (DBS-SCI): Protocol of a prospective one-armed multi-centre study. BMJ Open 2021;11(9):e047670.

2. Werner C, Easthope CA, Curt A, Demkó L. Towards a mobile gait analysis for patients with a spinal cord injury: A robust algorithm validated for slow walking speeds. Sensors 2021;21(21):7381.

3. Werner C, Gönel M, Lerch I, et al. Data-driven characterization of walking after a spinal cord injury using inertial sensors. Journal of NeuroEngineering and Rehabilitation 2023;20(1):55.

4. Balasubramanian S, Melendez-Calderon A, Roby-Brami A, Burdet E. On the analysis of movement smoothness. Journal of NeuroEngineering and Rehabilitation 2015;12:112.

5. Ackerman P, Morrison S, McDowell S, Vazquez L. Using the Spinal Cord Independence Measure III to measure functional recovery in a post-acute spinal cord injury program. Spinal Cord 2010;48(5):380–387.

6. Valentino R, Page M, Luppi P, et al. Evidence for widespread afferents to Barrington’s nucleus, a brainstem region rich in corticotropin-releasing hormone neurons. Neuroscience 1994;62(1):125–143.

7. Hofer A-S, Scheuber MI, Sartori AM, et al. Stimulation of the cuneiform nucleus enables training and boosts recovery after spinal cord injury. Brain 2022;145(10):3681–3697.

8. Hubscher CH, Herrity AN, Williams CS, et al. Improvements in bladder, bowel and sexual outcomes following task-specific locomotor training in human spinal cord injury. PLOS ONE 2018;13(1):1–26.

9. Haab F, Richard F, Amarenco G, et al. Comprehensive Evaluation of Bladder and Urethral Dysfunction Symptoms: Development and Psychometric Validation of the Urinary Symptom Profile (USP) Questionnaire. Urology 2008;71(4):646–656.

10. Krogh K, Christensen P, Sabroe S, Laurberg S. Neurogenic bowel dysfunction score. Spinal Cord 2006;44(10):625–631.
