# Supplementary figures and images for: Cuneiform Nucleus Stimulation Can Assist Gait Training to Promote Locomotor Recovery in Individuals With Incomplete Tetraplegia
Source: Ann Neurol. 2025 Sep 10;99(1):161–77. doi: 10.1002/ana.78026 (PMC12946608; doi:10.1002/ana.78026)

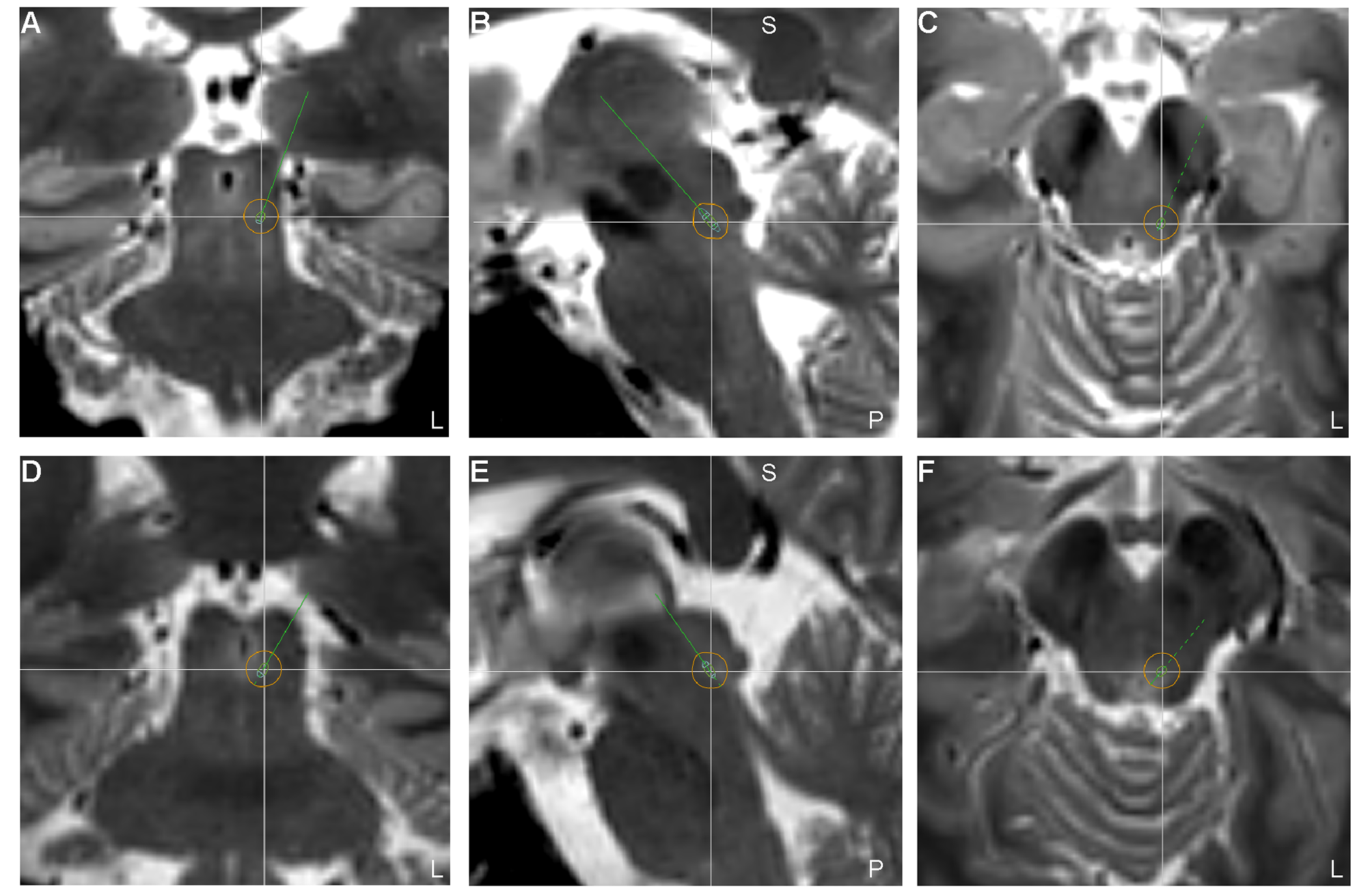

Supplement: Supplementary file 2 — Supplementary FIGURE S1: Stimulation volume modeling. [file ANA-99-161-s012.tif]

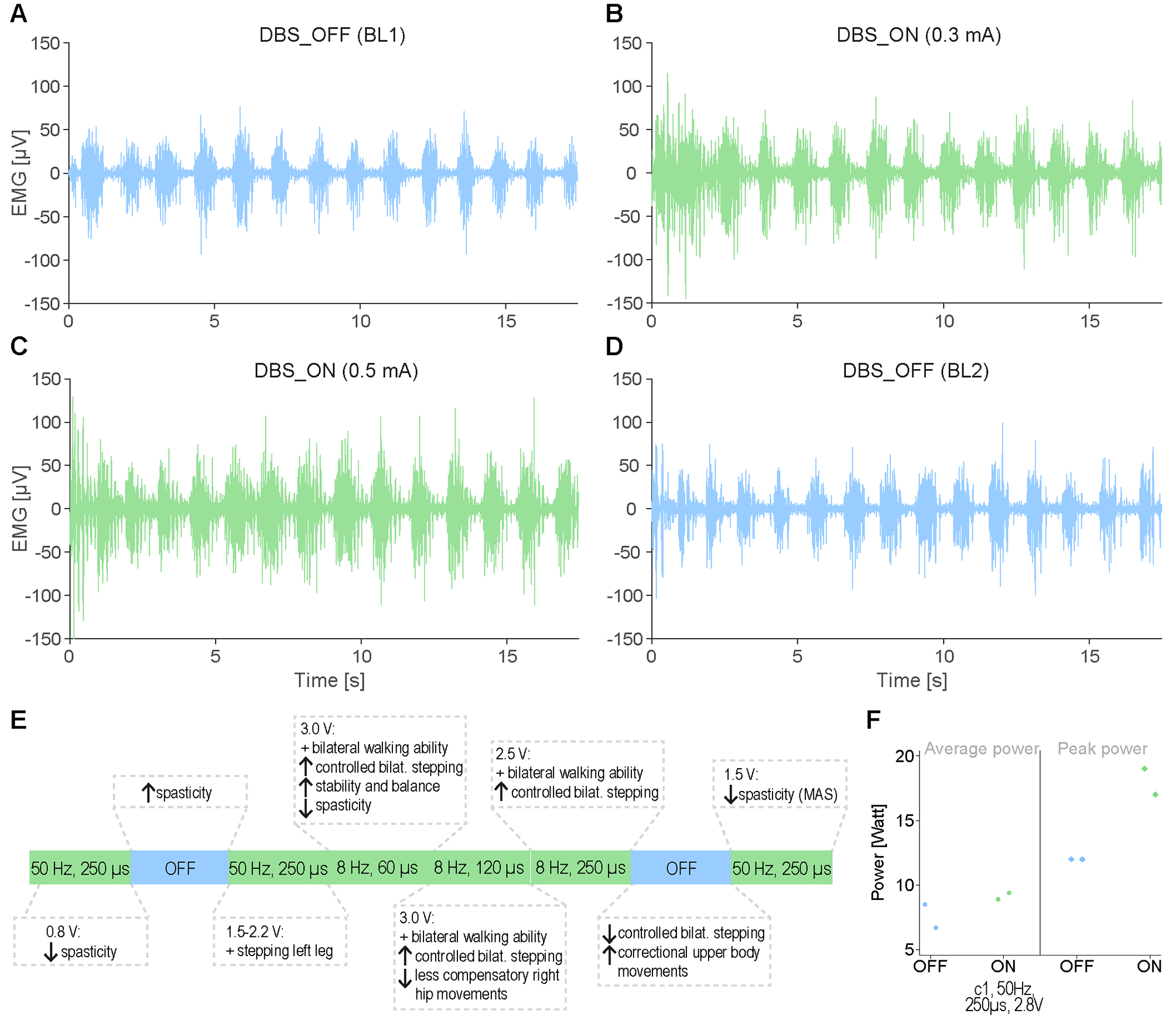

Supplement: Supplementary file 3 — Supplementary FIGURE S2: Intraoperative EMG and early postoperative stimulation effects in P1. [file ANA-99-161-s003.tif]

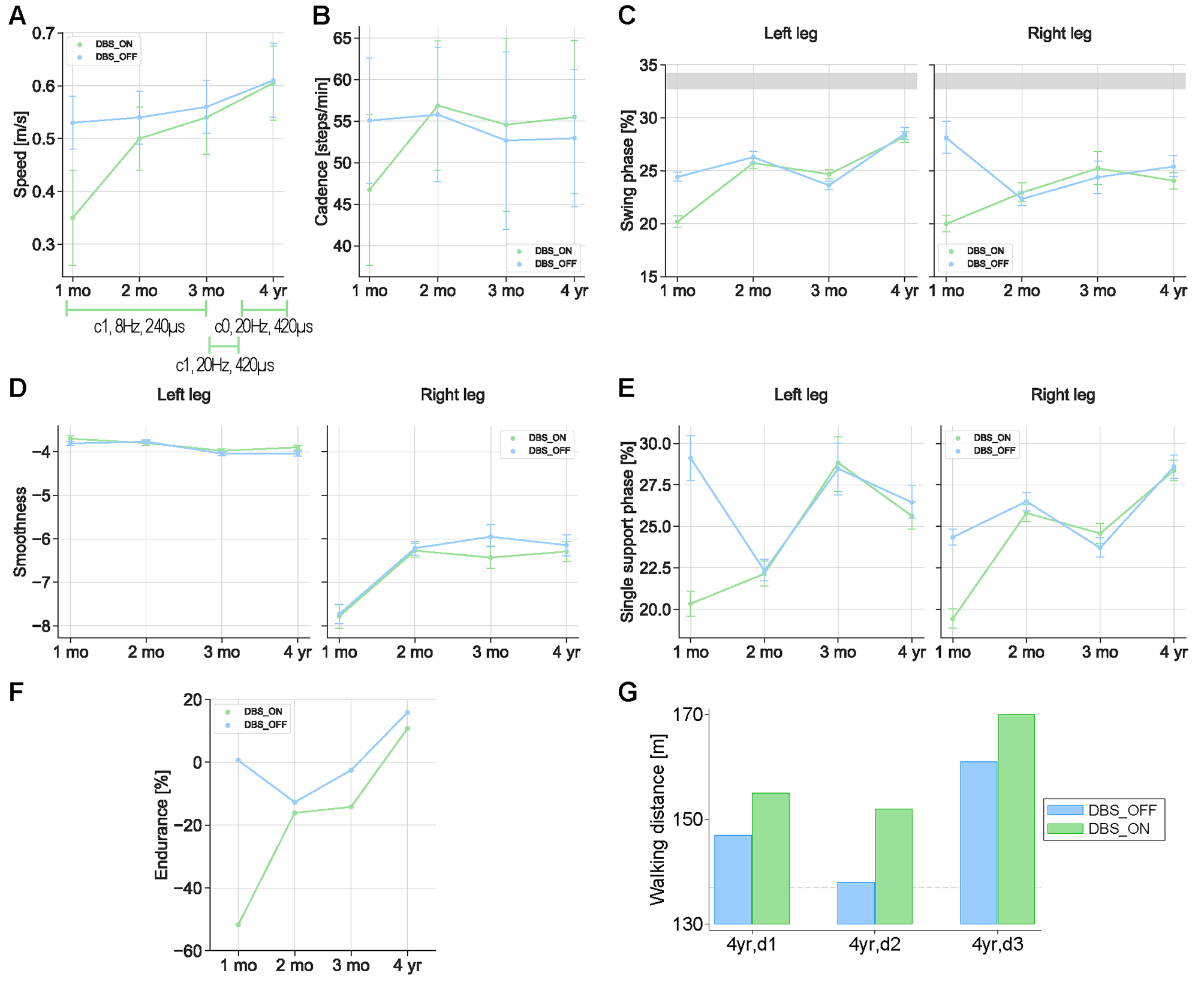

Supplement: Supplementary file 4 — Supplementary FIGURE S3: Sensor‐based gait analysis of P1 during selected 6‐Minute Walking Tests. [file ANA-99-161-s007.tif]

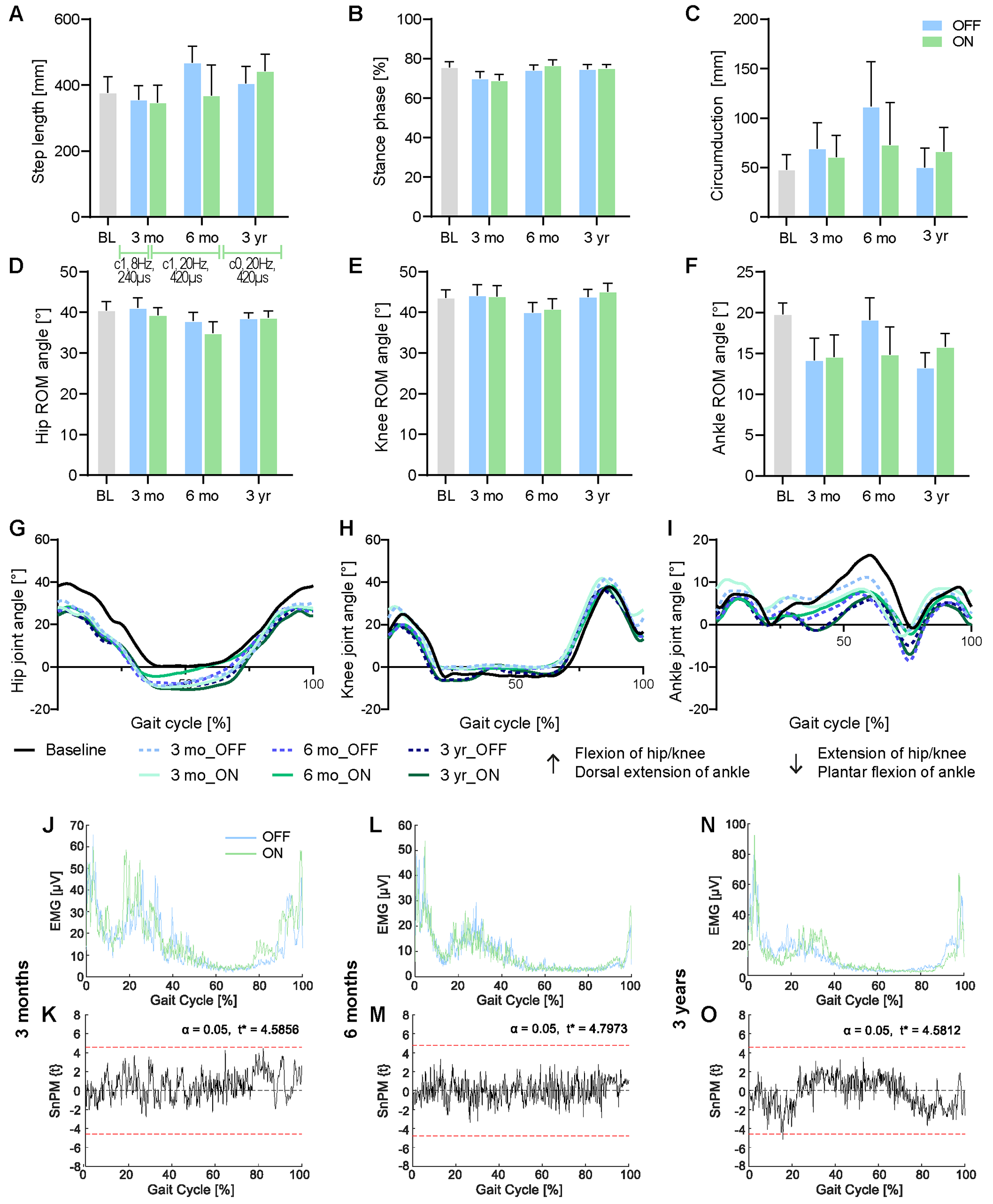

Supplement: Supplementary file 5 — Supplementary FIGURE S4: Left‐leg kinematic parameters during overground locomotion of P1. [file ANA-99-161-s013.tif]

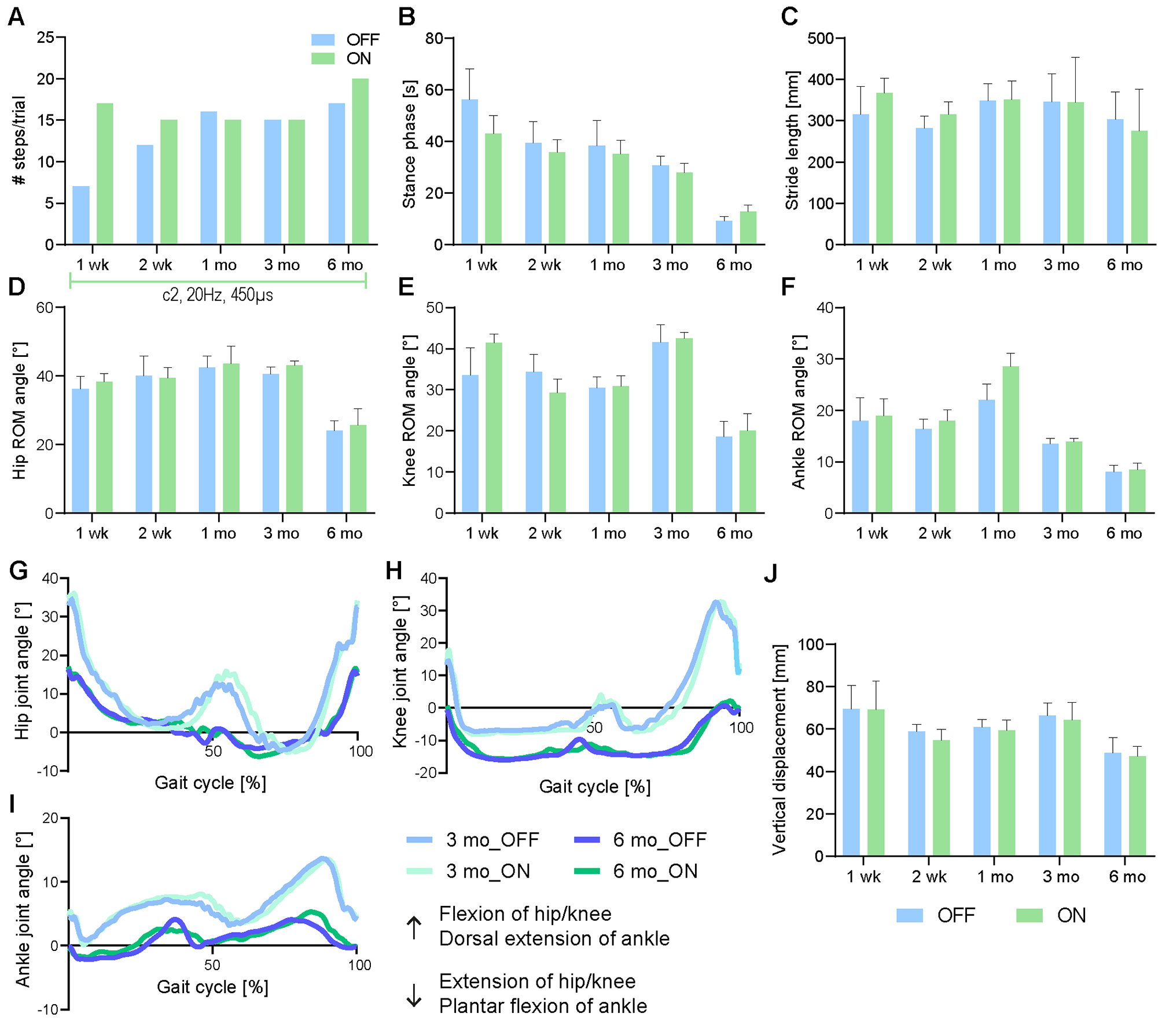

Supplement: Supplementary file 6 — Supplementary FIGURE S5: Left‐leg kinematic parameters during overground locomotion of P2. [file ANA-99-161-s011.tif]
