# Supplementary material for: Cuneiform Nucleus Stimulation Can Assist Gait Training to Promote Locomotor Recovery in Individuals With Incomplete Tetraplegia
Source: Ann Neurol. 2025 Sep 10;99(1):161–77. doi: 10.1002/ana.78026 (PMC12946608; doi:10.1002/ana.78026)
Supplement: Supplementary file 7 — Supplementary Table S1. Motor and sensory scores. [file ANA-99-161-s002.docx]

|  | **Patient 1** | | **Patient 2** | |
| --- | --- | --- | --- | --- |
|  | Baseline | 6-months | Baseline | 6-months |
| ASIA Impairment Scale (AIS)^a^ | D | D | C | C |
| Lower extremity motor score (LEMS)^b^ |  |  |  |  |
| Total |  |  |  |  |
| Left | 14 | 15 | 8 | 8 |
| Right | 6 | 6 | 4 | 4 |
| L2 (Hip flexors) |  |  |  |  |
| Left | 4 | 4 | 3 | 2 |
| Right | 0 | 0 | 3 | 2 |
| L3 (Knee extensors) |  |  |  |  |
| Left | 4 | 4 | 0 | 1 |
| Right | 4 | 4 | 0 | 1 |
| L4 (Ankle dorsiflexors) |  |  |  |  |
| Left | 2 | 2 | 0 | 0 |
| Right | 0 | 0 | 0 | 0 |
| L5 (Long toe extensors) |  |  |  |  |
| Left | 1 | 2 | 2 | 2 |
| Right | 0 | 0 | 0 | 0 |
| S1 (Ankle plantar flexors) |  |  |  |  |
| Left | 3 | 3 | 3 | 3 |
| Right | 2 | 2 | 1 | 1 |
| Lower extremity sensory score (L1 to S4-5)^c^ |  |  |  |  |
| Light touch |  |  |  |  |
| Left | 16 | 18 | 9 | 9 |
| Right | 18 | 18 | 9 | 9 |
| Pin prick |  |  |  |  |
| Left | 0 | 0 | 8 | 4 |
| Right | 18 | 18 | 7 | 4 |
| Spinal Cord Independence Measure (SCIM) III^d^ | 70 | 69 | 42 | 42 |
| Walking Index for Spinal Cord Injury (WISCI) II^e^ | 9 | 9 | 3 | 3 |
| Electrophysiological measurements^f^ |  |  |  |  |
| Lumbar tibialis anterior MEPs [ms/µV] |  |  |  |  |
| Left | 12.0/33 | 11.4/68 | 13.8/150 | 12.2/210 |
| Right | 12.0/36 | 11.7/20 | 14.3/130 | 13.8/220 |
| Cortical tibialis anterior MEPs [ms/µV] |  |  |  |  |
| Left | 38.5/200 | n.m. | NA/0 | n.m. |
| Right | NA/0 | n.m. | NA/0 | n.m. |
| Tibialis SSEPs [ms/µV] |  |  |  |  |
| Left | 35.8/0.8 | 35.3/0.25 | NA/0 | 61.6/0.48 |
| Right | 45.8/0.9 | 42.6/1.82 | NA/0 | 60.6/1.30 |

**Table S1. Motor and sensory scores.** ^a^AIS: A = Functionally complete spinal cord injury (SCI); B = Functionally sensory incomplete, motor complete SCI; C = Functionally motor incomplete (± some preservation of sensory function); D = Motor incomplete with ≥50% of key muscle function with muscle grade ≥3; E = Intact motor and sensory function. ^b^LEMS: Score range of 0 to 50; maximum of 5 for each of 5 key muscles of each leg. ^c^Lower extremity sensory score: only the sensory scores of the lower extremities = L1 to S4-5 are depicted (max. 2 points for each of 9 key sensory points per leg and body side; maximum score of 18 for light touch and pin prick). ^d^SCIM III: total of 19 items, subdivided into 3 subscales self-care, respiration and sphincter management, and mobility; total score = 100, subscales are weighted as follows: self-care - scored 0-20, respiration and sphincter management - scored 0-40, mobility - scored 0-40; the higher the score, the less assistance or fewer aids are required to complete basic activities of daily living. ^e^WISCI II: Score ranging from 0 to 20 (0 = patient unable to stand and/or participate in assisted walking; 20 = patient ambulates for 10m with no devices, no braces, and requires no physical assistance). ASIA = American Spinal Injury Association. ^f^Electrophysiology: MEP = motor evoked potentials. SSEPs = somatosensory evoked potentials. ms = latency. µV = amplitude. NA = not applicable (absent). n.m. = cortical MEPs not measured after implantation for safety reasons.
