# Supplementary material for: Cuneiform Nucleus Stimulation Can Assist Gait Training to Promote Locomotor Recovery in Individuals With Incomplete Tetraplegia
Source: Ann Neurol. 2025 Sep 10;99(1):161–77. doi: 10.1002/ana.78026 (PMC12946608; doi:10.1002/ana.78026)
Supplement: Supplementary file 8 — Supplementary Table S2. Overview of side‐effect thresholds and subjective motor observations in dependence on stimulation parameters and contacts during DBS‐testing in patient 1. [file ANA-99-161-s005.docx]

| **Frequency [Hz]** | **Pulse width [µs]** | **Contact** | **Side-effect TH [V]** | **Side-effect** | **Motor observations** |
| --- | --- | --- | --- | --- | --- |
| 8 | 60 | 1-C+ | 1.6 | Right-sided oscillopsia |  |
| 8 | 120 | 1-C+ | 1.2 | Right-sided oscillopsia |  |
| 8 | 240 | 1-C+ | 1.0 | Right-sided oscillopsia | 1.0 V: Subjectively good feeling, walking without intermediate steps |
| 8 | 240 | 2-C+ | 1.3 | Right-sided oscillopsia |  |
| 8 | 420 | 1-C+ | 0.8 | Right-sided oscillopsia |  |
| 20 | 60 | 1-C+ | 1.4 | Right-sided oscillopsia |  |
| 20 | 120 | 1-C+ | 1.1 | Right-sided oscillopsia | 1.1 V: Coordinated walking (right>left) |
| 20 | 240 | 1-C+ | 0.9 | Right-sided oscillopsia |  |
| 20 | 240 | 2-C+ | 1.3 | Right-sided oscillopsia |  |
| 20 | 420 | 0-C+ | 0.8 | Transient right-sided upper inner quadrant oscillopsia, self-limited tingling in right hand |  |
| 20 | 420 | 1-C+ | 0.8 | Right-sided oscillopsia | 0.8 V: Subjectively good feeling, coordinated walking |
| 20 | 420 | 0-1-C+ | 0.7 | Right-sided oscillopsia |  |
| 20 | 450 | 0-C+ | 0.8 | Wave-like right-sided oscillopsia during left-upwards gaze |  |
| 40 | 60 | 1-C+ | 1.7 | Right-sided oscillopsia |  |
| 40 | 120 | 1-C+ | 1.2 | Right-sided oscillopsia |  |
| 40 | 240 | 1-C+ | 1.1 | Right-sided oscillopsia |  |
| 40 | 240 | 2-C+ | 1.5 | Right-sided oscillopsia | 1.2 V: Feeling of restlessness, back muscle contractions |
| 40 | 420 | 1-C+ | 1.0 | Right-sided oscillopsia |  |
| 50 | 60 | 1-C+ | 1.9 | Transient feeling of tiredness/dizziness when looking into bright light |  |
| 50 | 120 | 1-C+ | 1.6 | Photophobia |  |
| 50 | 240 | 1-C+ | 1.2 | Photophobia, feeling of tiredness/dizziness | 1.0 V: Tingling sensation in back |
| 50 | 240 | 2-C+ | 1.4 | Photophobia | 1.3 V: Feeling of restlessness, back muscle contractions |
| 50 | 240 | 1-2-C+ | 0.9 | Right-sided oscillopsia |  |
| 50 | 420 | 1-C+ | 1.1 | Photophobia, cold sensation in face |  |

**Table S2. Overview of side-effect thresholds and subjective motor observations in dependence on stimulation parameters and contacts during DBS-testing in patient 1.** TH = threshold.
