# Supplementary material for: Cuneiform Nucleus Stimulation Can Assist Gait Training to Promote Locomotor Recovery in Individuals With Incomplete Tetraplegia
Source: Ann Neurol. 2025 Sep 10;99(1):161–77. doi: 10.1002/ana.78026 (PMC12946608; doi:10.1002/ana.78026)
Supplement: Supplementary file 9 — Supplementary TABLE S3. Training schedule during inpatient rehabilitation at Balgrist University Hospital. [file ANA-99-161-s008.docx]

| **TR week** | **Study month** | **PT** | **LM** | **MM** | **Stand** | **Walk** | **MTT** | **TM** | **B/HC** | **H2O** | **OT** | **TT** |
| --- | --- | --- | --- | --- | --- | --- | --- | --- | --- | --- | --- | --- |
|  | **Patient 1** | | | | | | | | | | | |
| 1 | 1 | 1 | 0 | 0 | 0 | 1 | 0 | 1 | 0 | 0 | 0 | 3 |
| 2 | 1 | 2 | 0 | 0 | 0 | 2 | 1 | 2 | 0 | 0 | 1.5 | 8.5 |
| 3 | 1 | 0 | 0 | 1.5 | 0 | 3 | 4.5 | 6 | 1.5 | 0 | 2 | 18.5 |
| 4 | 1 | 0 | 0 | 0.5 | 0 | 0 | 0 | 4 | 0 | 0 | 1.5 | 6 |
| 5 | 2 | 0 | 2 | 0.5 | 0 | 1 | 1.5 | 3 | 0.5 | 0 | 1.5 | 10 |
| 6 | 2 | 0 | 0 | 0 | 0 | 3 | 0 | 2 | 0 | 0 | 2 | 7 |
| 7-10 | 2-3 | external inpatient rehabilitation | | | | | | | | | | |
|  |  | 2 | 0 | 0 | 2 | 5 | 0 | 5 | 3 | 0 | 0 | 17 |
| 11-19 | 3-5 | self-training at home | | | | | | | | | | |
|  |  | 2 | 0 | 0 | 2 | 5 | 0 | 0 | 0 | 1 | 0 | 10 |
| 20-24 | 5-6 | external inpatient rehabilitation | | | | | | | | | | |
|  |  | 2 | 0 | 0 | 2 | 5 | 0 | 5 | 3 | 0 | 0 | 17 |
|  | **Patient 2** | | | | | | | | | | | |
| 1 | 1 | 5 | 1 | 0 | 0 | 0 | 0 | 0 | 0 | 0 | 0 | 6 |
| 2 | 1 | 2 | 2 | 0.5 | 1.5 | 2 | 0.5 | 1 | 0 | 0 | 0 | 9.5 |
| 3 | 1 | 2.5 | 1 | 0 | 0.5 | 1 | 0 | 0.5 | 0 | 0 | 0 | 5.5 |
| 4 | 2 | 2 | 1 | 0 | 0 | 2 | 0 | 1 | 0 | 0 | 0 | 6 |
| 5-10 | 2-3 | irregular self-training at home | | | | | | | | | | |
| 11 | 3 | 4.5 | 1 | 2 | 0 | 0 | 3 | 0 | 1 | 0 | 0 | 11.5 |
| 12 | 3 | 2.5 | 2 | 2 | 0 | 0 | 3 | 0 | 0 | 0 | 0 | 9.5 |
| 13 | 4 | 4.5 | 2 | 1.5 | 0 | 0 | 3.5 | 0 | 0 | 0 | 0 | 11.5 |
| 14 | 4 | 3.5 | 2 | 2.5 | 0 | 0 | 2 | 0 | 0.5 | 0.75 | 0 | 11.25 |
| 15 | 4 | 5.25 | 2 | 2.5 | 0 | 0 | 2 | 0 | 1 | 0 | 0 | 12.75 |
| 16 | 4 | 4.25 | 2 | 1.5 | 0 | 0 | 2 | 0 | 1 | 0 | 0 | 10.75 |
| 17 | 5 | 3 | 0 | 1.5 | 0 | 1 | 2 | 0 | 0.5 | 0 | 0 | 8 |
| 18 | 5 | 3.5 | 2 | 2 | 0 | 1.75 | 3 | 0 | 1.5 | 0 | 0 | 13.75 |
| 19 | 5 | 3 | 3 | 1.5 | 0 | 1 | 2 | 0 | 1 | 0 | 0 | 11.5 |
| 20-24 | 5-6 | discontinued self-training at home | | | | | | | | | | |

**Table S3. Training schedule during inpatient rehabilitation at Balgrist University Hospital**. TR = training. PT = physiotherapy. LM = Lokomat (body weight support, patient 1: 0%; patient 2: on average 50%). MM = Motomed. Stand = Standing. Walk = overground gait training (body weight support, patient 1: 0%; patient 2: 0% in parallel bars, on average 15% in FLOAT). MTT = medical training therapy. TM = treadmill (body weight support, patient 1: 10%; patient 2: on average 15%). B/HC = bicycle (patient 1)/handcycle (patient 2). H2O = hydrotherapy. OT = occupational therapy. TT = Training in total. Amount of training is given in hours.
