# Supplementary material for: Cuneiform Nucleus Stimulation Can Assist Gait Training to Promote Locomotor Recovery in Individuals With Incomplete Tetraplegia
Source: Ann Neurol. 2025 Sep 10;99(1):161–77. doi: 10.1002/ana.78026 (PMC12946608; doi:10.1002/ana.78026)
Supplement: Supplementary file 10 — Supplementary TABLE S4. Overview of side‐effect thresholds and subjective motor observations in dependence on stimulation parameters and contacts during DBS‐testing in patient 2. [file ANA-99-161-s009.docx]

| **Frequency [Hz]** | **Pulse width [µs]** | **Contact** | **Side-effect TH [V]** | **Side-effect** | **Motor observations** |
| --- | --- | --- | --- | --- | --- |
| 20 | 450 | 0-C+ | 0.9 | Bilateral oscillopsia  (left: lower half; right: upper half) |  |
| 20 | 450 | 1-C+ | 0.9 | Ipsilateral oscillopsia |  |
| 20 | 450 | 2-C+ | 1.2 | Ipsilateral oscillopsia,  ipsilateral saccadic eye movements | 0.2 V: convenient feeling in both legs, improved gait |
| 20 | 450 | 3-C+ | 1.5 | Ipsilateral oscillopsia |  |
| 20 | 450 | 1-2-C+ | 1.5 | Left-sided lagophthalmos | 1.5 V: subjectively improved gait |
| 8 | 450 | 0-C+ | 0.8 | Low frequency contralateral oscillopsia |  |
| 8 | 450 | 1-C+ | 1.2 | Low frequency bilateral oscillopsia |  |
| 8 | 450 | 2-C+ | 1.6 | Bilateral low-frequency oscillopsia, slow saccadic eye movements |  |
| 8 | 450 | 3-C+ | 1.6 | Low frequency ipsilateral oscillopsia |  |
| 50 | 450 | 0-C+ | 1.0 | Double vision | 1.0 V: Convenient feeling in both legs, increased spasticity |
| 50 | 450 | 1-C+ | 1.1 | Blurred vision (trouble focusing, oculomotor function intact) | 1.0 V: Convenient feeling in both legs, increased spasticity |
| 50 | 450 | 2-C+ | 1.3 | Blurred vision (trouble focusing, oculomotor function intact) | 0.7 V: Convenient feeling in both legs (left>right, distal>proximal), increased spasticity |
| 50 | 450 | 3-C+ | 1.7 | Double vision | 1.3 V: Convenient abdominal/leg muscle tension, increased spasticity |
| 50 | 450 | 1-2-C+ | 1.0 | Left-sided lagophthalmos,  spasticity hindering walking | 0.9 V: Convenient feeling in both legs, abdominal muscle tension, increased spasticity |

**Table S4. Overview of side-effect thresholds and subjective motor observations in dependence on stimulation parameters and contacts during DBS-testing in patient 2.** TH = threshold.
