# Supplementary material for: Cuneiform Nucleus Stimulation Can Assist Gait Training to Promote Locomotor Recovery in Individuals With Incomplete Tetraplegia
Source: Ann Neurol. 2025 Sep 10;99(1):161–77. doi: 10.1002/ana.78026 (PMC12946608; doi:10.1002/ana.78026)
Supplement: Supplementary file 11 — Supplementary TABLE S5. Modified Ashworth Scale (MAS) of spasticity. [file ANA-99-161-s001.docx]

|  | **Patient 1** | | | | | **Patient 2** | | | | |
| --- | --- | --- | --- | --- | --- | --- | --- | --- | --- | --- |
|  | BL | 1wk | 1mo | 3mo | 6mo | BL | 1wk | 1mo | 3mo | 6mo |
| Shoulder |  |  |  |  |  |  |  |  |  |  |
| Left | 0 | 0 | 0 | 0 | 0 | 0 | 0 | 0 | 0 | 0 |
| Right | 0 | 0 | 0 | 0 | 0 | 0 | 0 | 0 | 0 | 0 |
| Elbow |  |  |  |  |  |  |  |  |  |  |
| Left | 0 | 0 | 0 | 0 | 0 | 0 | 0 | 0 | 0 | 0 |
| Right | 0 | 0 | 0 | 0 | 0 | 0 | 0 | 0 | 0 | 0 |
| Wrist |  |  |  |  |  |  |  |  |  |  |
| Left | 0 | 0 | 0 | 1 | 1 | 0 | 0 | 0 | 0 | 0 |
| Right | 0 | 0 | 0 | 1 | 0 | 0 | 0 | 0 | 0 | 0 |
| Fingers |  |  |  |  |  |  |  |  |  |  |
| Left | 0 | 0 | 0 | 1 | 1 | 1+ | 0 | 1+ | 1+ | 2 |
| Right | 1+ | 1+ | 1+ | 1 | 1+ | 2 | 0 | 1+ | 1+ | 2 |
| Hip |  |  |  |  |  |  |  |  |  |  |
| Left | **2** | 2 | 2 | 2 | **0** | 3 | 3 | 3 | 2 | 3 |
| Right | 2 | 2 | 2 | 2 | 1 | 3 | 3 | 3 | 3 | 3 |
| Knee |  |  |  |  |  |  |  |  |  |  |
| Left | **2** | 2 | 2 | 2 | **0** | 3 | 3 | 3 | 2 | 3 |
| Right | 2 | 2 | 3 | 3 | 3 | 3 | 3 | 3 | 3 | 3 |
| Ankle |  |  |  |  |  |  |  |  |  |  |
| Left | 3 | 3 | 3 | 3 | 3 | 4 | 4 | 4 | 2 | 4 |
| Right | 3 | 3 | 3 | 3 | 3 | 4 | 4 | 4 | 3 | 4 |

**Table S5. Modified Ashworth Scale (MAS)^a^ of spasticity.** ^a^MAS: Scores range from 0 (no increase in tone) to 4 (affected part in rigid flexion and extension). BL = baseline. 1wk = 1-week timepoint. 1mo = 1-month timepoint. 3mo = 3-months timepoint. 6mo = 6-months timepoint. Values highlighted in bold indicate relevant improvement.
