# Supplementary material for: Cuneiform Nucleus Stimulation Can Assist Gait Training to Promote Locomotor Recovery in Individuals With Incomplete Tetraplegia
Source: Ann Neurol. 2025 Sep 10;99(1):161–77. doi: 10.1002/ana.78026 (PMC12946608; doi:10.1002/ana.78026)
Supplement: Supplementary file 12 — Supplementary TABLE S6. Lower urinary tract and bowel function. [file ANA-99-161-s010.docx]

| **Autonomic function** | **Patient 1** | | | | **Patient 2** | | | |
| --- | --- | --- | --- | --- | --- | --- | --- | --- |
|  | **Scr** | | **6mo** | | **Scr** | | **6mo** | |
| **Lower urinary tract function** |  | |  | |  | |  | |
| Qualiveen^a^ |  | |  | |  | |  | |
| Bother with limitations | 0.0 | | 1.1 | | 1.0 | | 1.2 | |
| Frequency of limitations | 1.1 | | 2.0 | | 2.5 | | 2.0 | |
| Fears | 0.8 | | 0.5 | | 1.5 | | 0.6 | |
| Feeling | 1.8 | | 1.2 | | 1.2 | | 1.2 | |
| Total | 0.9 | | 1.2 | | 1.6 | | 1.3 | |
| Bladder diary |  | |  | |  | |  | |
| Average voided volume [ml] | 195(90-330) | | 185(100-270) | | 230(100-400) | | NA | |
| Daytime frequency | 8-13 | | 7-10 | | 0-6 | | 0 | |
| Average catheterized volume [ml] | NA | | NA | | 455(200-700) | | 335(150-750) | |
| Catheter frequency | 0 | | 0 | | 4-6 | | 9-11 | |
| Average fluid intake [ml/24h] | 2150 | | 1400 | | 2135 | | 2300 | |
| Free uroflowmetry |  | |  | |  | |  | |
| Qmax [ml/s] | **12** | | **9** | | NA | | NA | |
| Voided volume [ml] | **115** | | **80** | | NA | | NA | |
| Postvoid residual volume [ml] | **80** | | **0** | | NA | | NA | |
| Urinary symptom profile^b^ |  | |  | |  | |  | |
| Stress incontinence score | NA | | NA | | **9** | | **0** | |
| Overactive bladder score | NA | | NA | | **18** | | **6** | |
| Low stream score | NA | | NA | | **4** | | **9** | |
| **Bowel function** |  | |  | |  | |  | |
| Neurogenic Bowel Dysfunction score^c^ | NA | | NA | | **15** | | **11** | |
| **Video-urodynamic investigation** | **Scr** | **6mo OFF** | | **6mo**  **ON** | **Scr** | **6mo OFF** | | **6mo ON** |
| Filling cystometry |  |  | |  |  |  | |  |
| Max. cystometric capacity [ml] | 160 | 75 | | 115 | 440 | 590 | | 590 |
| Compliance [ml/cmH_2_O] | 34 | 35 | | 47 | 47 | 56 | | 51 |
| Detrusor overactivity |  |  | |  |  |  | |  |
| Present | Yes | Yes | | Yes | Yes | Yes | | Yes |
| Start volume [ml] | 85 | **75** | | **95** | 320 | **300** | | **390** |
| Incontinence | Yes | Yes | | Yes | Yes | Yes | | Yes |
| Max. detrusor storage pressure | 66 | 65 | | 61 | 45 | 33 | | 32 |
| Pressure flow |  |  | |  |  |  | |  |
| DSD | Yes | Yes | | Yes | Yes | Yes | | Yes |
| Spontaneous/reflex voiding | Yes | Yes | | Yes | Yes | Yes | | Yes |
| Max. flow rate [ml/s] | 14 | 9 | | 5 | 8 | 4 | | 1 |
| Detrusor pressure, max. flow rate [cmH_2_O] | 47 | 44 | | 44 | 26 | 24 | | 19 |
| Max. detrusor pressure, voiding [cmH_2_O] | 66 | 65 | | 61 | 44 | 33 | | 32 |
| Voided/leaked volume [ml] | 160 | 75 | | 115 | 110 | 40 | | 50 |
| Postvoid residual volume [ml] | 0 | 0 | | 0 | 330 | 550 | | 540 |
| Vesico-uretero-renal reflux | No | No | | No | No | No | | No |
| Autonomic dysreflexia | No | No | | No | No | No | | No |

**Table S6. Lower urinary tract and bowel function.** ^a^Qualiveen: 0 (no impact) – 4 (high adverse impact); the lower the score the better quality of life. ^b^Urinary Symptom Profile (USP): stress incontinence score – scored 0-9; overactive bladder score – scored 0-21; low stream score – scored 0-9; the higher the score the worse symptoms. ^c^Neurogenic Bowel Dysfunction (NBD) score: 0-6 = very minor dysfunction; 7-9 = minor dysfunction; 10-13 = moderate dysfunction; ≥14 = severe dysfunction. Scr = Screening. 6mo = 6-months timepoint. DSD = detrusor sphincter dyssynergia; NA = not applicable; Max. = maximum; OFF = without stimulation; ON = with stimulation. Qmax = maximum flow rate. Values highlighted in bold indicate relevant functional improvement.
