# Supplementary material for: Cuneiform Nucleus Stimulation Can Assist Gait Training to Promote Locomotor Recovery in Individuals With Incomplete Tetraplegia
Source: Ann Neurol. 2025 Sep 10;99(1):161–77. doi: 10.1002/ana.78026 (PMC12946608; doi:10.1002/ana.78026)
Supplement: Supplementary file 13 — Supplementary TABLE S7. Scoring of sexual function, sleepiness, fatigue, pain, and quality of life. [file ANA-99-161-s006.docx]

|  | **Patient 1** | | | | **Patient 2** | | | |
| --- | --- | --- | --- | --- | --- | --- | --- | --- |
|  | BL | 1mo | 3mo | 6mo | BL | 1mo | 3mo | 6mo |
| FSFI^a^ | 2.9 | NA* | NA* | 3.6 | NA | NA | NA | NA |
| IIEF^b^ | NA | NA | NA | NA | 14 | 14 | 16 | 14 |
| ESS^c^ | 3 | 6 | 10 | 11 | 6 | 4 | 1 | 2 |
| FSS^d^ | 3.1 | 4.3 | 4.7 | 4.8 | 3 | 2.6 | 1.56 | 3.4 |
| SCIPI^e^ | 3 | 0 | 1 | 1 | 2 | 4 | 2 | 2 |
| SF-36^f^ [%] |  |  |  |  |  |  |  |  |
| Physical functioning | 15 | 25 | 25 | 30 | 0 | 10 | 10 | 5 |
| Role limitation (physical) | 100 | 25 | 25 | 50 | 0 | 100 | 100 | 100 |
| Role limitation (emotional) | 100 | 100 | 100 | 100 | 33.3 | 100 | 100 | 66.7 |
| Energy/Fatigue | 50 | 60 | 60 | 50 | 60 | 50 | 60 | 55 |
| Emotional well-being | 60 | 92 | 92 | 96 | 60 | 68 | 52 | 76 |
| Social functioning | 100 | 87.5 | 87.5 | 87.5 | 62.5 | 87.5 | 50 | 75 |
| Pain | 100 | 100 | 100 | 90 | 70 | 80 | 57.5 | 70 |
| General health | 60 | 80 | 80 | 85 | 70 | 85 | 80 | 80 |
| Health change | 75 | 75 | 75 | 75 | 50 | 50 | 75 | 50 |

**Table S7. Scoring of sexual function, sleepiness, fatigue, pain, and quality of life.** ^a^FSFI: maximum score: 36; higher score indicates better sexual functioning. ^b^IIEF: maximum score: 30; higher score indicates better sexual functioning. ^c^ESS: maximum score: 24; the higher the score the higher daytime sleepiness; cutoff for severe sleepiness: >14. ^d^FSS: maximum score: 7; the higher the score the higher level of fatigue. ^e^SCIPI: maximum score: 4; the higher the score the stronger the probability of SCI related neuropathic pain. ^f^SF-36: score from 0 to 100%; the higher the score the less health restrictions. BL = baseline. 1mo = 1-month timepoint. 3mo = 3-months timepoint. 6mo = 6-months timepoint. ESS = Epworth Sleepiness Scale. FSFI = Female Sexual Function Index. FSS = Fatigue Severity Scale. IIEF = International Index of Erectile Function. SCIPI = Spinal Cord Injury Pain Instrument. SF-36 = Short Form Health Survey to Assess Quality of Life. *Due to missing data in one subscore the total FSFI could not be calculated for the 1-month and the 3-months timepoint.
